# Tegotae-Based Control Produces Adaptive Inter- and Intra-limb Coordination in Bipedal Walking

**DOI:** 10.3389/fnbot.2021.629595

**Published:** 2021-05-12

**Authors:** Dai Owaki, Shun-ya Horikiri, Jun Nishii, Akio Ishiguro

**Affiliations:** ^1^Department of Robotics, Graduate School of Engineering, Tohoku University, Sendai, Japan; ^2^Research Institute of Electrical Communication, Tohoku University, Sendai, Japan; ^3^Graduate School of Sciences and Technology for Innovation, Yamaguchi University, Yamaguchi, Japan

**Keywords:** bipedal walking, central pattern generator, inter- and intra-limb coordination, Tegotae, adaptability

## Abstract

Despite the appealing concept of central pattern generator (CPG)-based control for bipedal walking robots, there is currently no systematic methodology for designing a CPG-based controller. To remedy this oversight, we attempted to apply the Tegotae approach, a Japanese concept describing how well a perceived reaction, i.e., sensory information, matches an expectation, i.e., an intended motor command, in designing localised controllers in the CPG-based bipedal walking model. To this end, we developed a Tegotae function that quantifies the Tegotae concept. This function allowed incorporating decentralised controllers into the proposed bipedal walking model systematically. We designed a two-dimensional bipedal walking model using Tegotae functions and subsequently implemented it in simulations to validate the proposed design scheme. We found that our model can walk on both flat and uneven terrains and confirmed that the application of the Tegotae functions in all joint controllers results in excellent adaptability to environmental changes.

## 1. Introduction

The human body is capable of astoundingly adaptive and versatile locomotion when faced with real-world constraints. For robots to possess similar capabilities, their bodies must have comparable degrees of freedom (DOFs) more significant than those implemented in existing designs. Most previously developed centralised approaches to improving humanoid locomotion (Hirai et al., 1998; Sakagami et al., 2002; Hirukawa et al., 2004; Kaneko et al., 2004; Hirose and Ogawa, 2007), where one centralised controller regulates each DOF to continually track the desired trajectory of each point in the robot's body. However, this centralised approach is not suitable for systems with relatively large DOFs, leading to increased computational cost and reduced adaptability to unpredictable environmental changes.

Alternatively, autonomous decentralised control has received considerable attention because it offers the flexibility required for a robot with many DOFs to coordinate its movement successfully. In fact, animals deftly coordinate the many DOFs of their bodies using distributed neural networks called central pattern generators (CPGs), which are responsible for generating rhythmic movements, particularly locomotion (Shik et al., 1966; Grillner, 1975, 1985). Such knowledge about animal locomotion has been referenced by various researchers to incorporate artificial CPGs into legged robots for generating highly adaptive locomotion (Taga et al., 1991; Taga, 1994, 1995; Kimura et al., 1999, 2007; Fukuoka et al., 2003; Tsujita et al., 2003; Aoi and Tsuchiya, 2005, 2006; Buchli et al., 2006; Ijspeert, 2008; Righetti and Ijspeert, 2008).

CPG-based bipedal walking control originated in the pioneering work done by Taga et al. (1991) and Taga (1994, 1995). In these studies, sensory information from the environment was fed back to a nervous system model to generate walking from the interaction between the nervous system model, musculoskeletal model, and environment (“Global Entrainment”). Aoi and Tsuchiya (2005) and Aoi and Tsuchiya (2006) focused on “phase resetting” (Schomburg et al., 1998), a feedback mechanism found in animals, to add gait stabilisation in CPG-based control models. Furthermore, the feedback law based on phase resetting is suitable for musculoskeletal models (Aoi et al., 2010), which are more similar to humans. For generating stable motion in a bipedal robot through entrainment between a controller and robot motion, Morimoto et al. (2006) modelled the controller of the robot as an oscillator and the motion phase based on the position and velocity information of the centre of pressure (CoP) in the lateral direction of the robot, to achieve stepping and walking motions. Nassour et al. (2014) developed a two-layer CPG model for walking control in a humanoid robot: a rhythm generator layer and pattern formation layer (Rybak et al., 2006; McCrea and Rybak, 2008). They also attempted to generate non-periodic motions using neuron models that generate various signals such as periodic and non-periodic signals as components. Quadrupedal robots have been studied more intensively due to their dynamic stability and variety of walking patterns: Kimura et al. (1999) and Fukuoka et al. (2003) proposed a model integrating CPG and reflex mechanisms to realise uneven terrain walking; Tsujita et al. (2003) implemented the phase resetting in a quadrupedal walking model to actualise a stable walking pattern; Buchli et al. (2006) proposed an adaptive frequency oscillator that learns the motion frequency adaptively and verified the generation of gait according to the body characteristics; In addition, a model that employs load information as sensory information and generates adaptive and diverse walking patterns has been proposed thus far (Maufroy et al., 2010; Fukuoka et al., 2015; Owaki and Ishiguro, 2017). However, there is currently no systematic methodology for designing a CPG-based controller, as each CPG-based model has been custom-designed for a specific practical situation.

To address this oversight, we attempted to construct a CPG-based bipedal walking model with a localised joint-controller design based on the Tegotae approach (Owaki et al., 2017; Kano et al., 2019), which is a Japanese concept that focuses on how well a perceived reaction matches an expectation. We quantified the Tegotae concept by creating the Tegotae function, which is the quantified product of what a localised controller wants to achieve and its resulting reaction. The Tegotae function allows the systematic design of decentralised controllers with localised sensory feedback. The feedback scheme allows the operation of each localised controller based on consistency between the generated action and perceived reaction. Specifically, the Tegotae function increases in the case of consistency and decreases in the case of inconsistency. Here, we show how the Tegotae approach can be implemented in a decentralised control scheme for bipedal walking robots and validates the system by evaluating its adaptability to environmental changes.

## 2. Bipedal Walking Model

### 2.1. Musculoskeletal Structure

**Figure 1:**
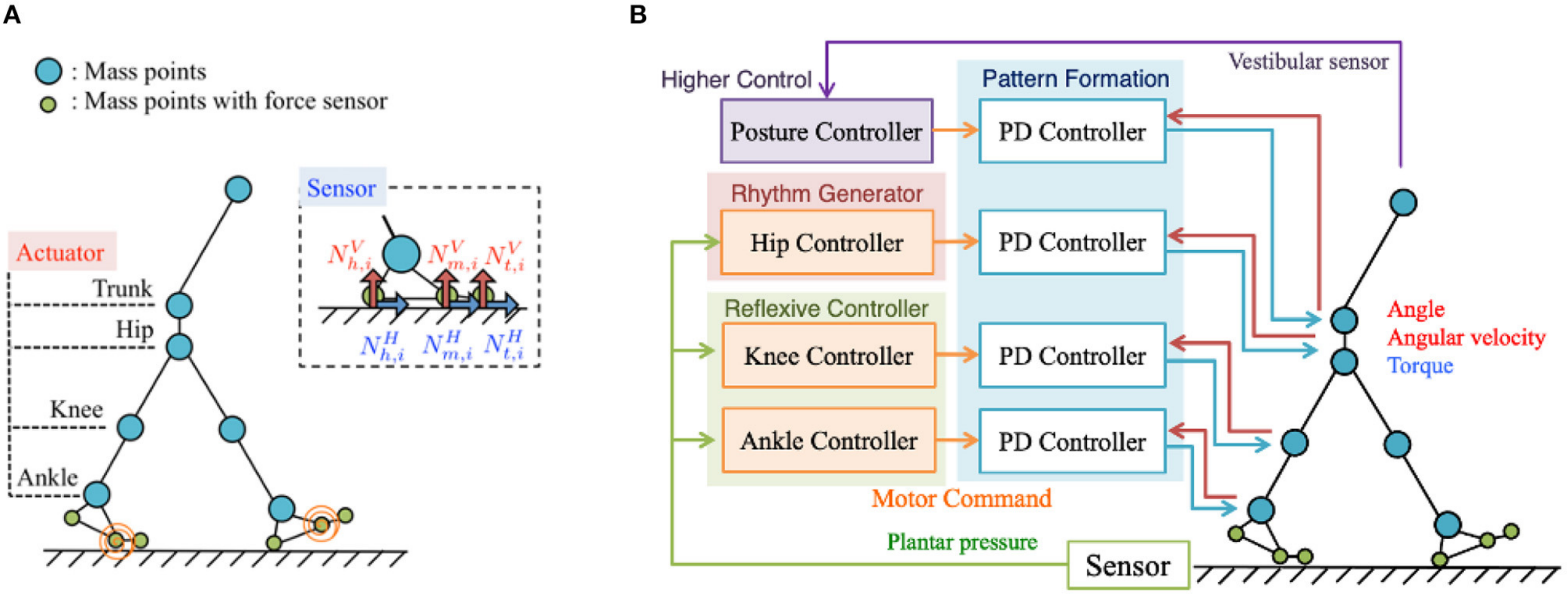
**(A)** Musculoskeletal structure of the bipedal walking model. For simplicity and ease of modelling, the masses are located in the joints and movements constrained in the sagittal plane. The structure consists of 13 mass points (i.e., the trunk, waist, hip, knees, ankles, heels, metatarsals, and toes) and 14 rigid links that connect these mass points. The plantar sensation is modelled by incorporating sensors to detect the vertical and horizontal ground reaction forces (GRFs) ($N_{x,i}^{V}$ and $N_{x,i}^{H}$, respectively) exerted at heel (*x* = *h*), metatarsal (*x* = *m*), and toe (*x* = *t*) points on the feet. **(B)** Control system overview. The proposed control system for adaptive bipedal walking consists of four main components: (i) hip controllers, (ii) knee controllers, (iii) ankle controllers, and (iv) a posture controller.

To validate the Tegotae-based control scheme, we conducted simulations using a two-dimensional bipedal walking model. Figure 1A shows the musculoskeletal structure of the bipedal walking model, the movements of which were constrained in the sagittal plane for simplicity. The structure consists of 13 mass points (i.e., the trunk, waist, hip, knees, ankles, heels, metatarsals, and toes) and 14 rigid links that connect these mass points. For simplicity and ease of modelling the musculoskeletal system, we employed a model with masses located in the joints. The body parameters, e.g., link length, mass distribution, were set to approximately match the corresponding human body parameters in Ogihara and Yamazaki (2001). The model includes seven actuators at the waist, hip joints, knee joints, and ankle joints; each actuator was designed to generate joint torque based on proportional-derivative (PD) control, as explained in section 2.2. Passive springs and dampers have been integrated into the toe joints to passively generate an effective push-off force at the end of the stance phase. Based on human and animal locomotion research, that show the role of cutaneous receptors in the foot in controlling the gait (Nurse and Nigg, 1999, 2001; Dietz and Duysens, 2000; Duysens et al., 2000; Eils et al., 2002; Elis et al., 2004), we modelled plantar sensation by incorporating sensors to detect the vertical and horizontal ground reaction forces (GRFs) ($N_{x,i}^{V}$ and $N_{x,i}^{H}$, respectively) exerted at heel (*x* = *h*), metatarsal (*x* = *m*), and toe (*x* = *t*) points. Here, the suffix *i* denotes leg (*i* = 0: left and *i* = 1: right). In this study, the equations of motion were constructed as dynamics of mass points. For each mass point, the following forces were applied: force due to gravity, force applied by the links modelled with a rigid spring and damper, force applied by the actuators of each joint, and force applied by the passive spring and damper at toe joints. The details are described in the Supplementary Material.

### 2.2. Implementation of Tegotae Approach in a Systematic CPG-Based Control Scheme

The proposed control system for adaptive bipedal walking consists of four components (Figure 1B): (1) hip controllers, (2) knee controllers, (3) ankle controllers, and (4) a posture controller. The first three components utilise Tegotae functions to coordinate the inter- and intra-limb movements to enable adaptive walking, whereas the fourth component stabilises the upper body using the waist actuator and vestibular sensor.

The hip, knee, ankle, trunk joint torques τ_*y, i*_ in each *ith* leg (*y* indicates one of the joints) are generated by the PD control mechanism, which is dependent on the target angles determined by the hip, knee, ankle, and posture controllers. These torques are calculated as follows:

(1) $$\begin{array}{r} \tau_{hip,i}=-K_{hip}\left(\theta_{hip,i}-{\bar{\theta }}_{hip,i}\right)-D_{hip}{\overset{∙}{\theta }}_{hip,i}, \end{array}$$

(2) $$\begin{array}{r} \tau_{knee,i}=-K_{knee,i}\left(\theta_{knee,i}-{\bar{\theta }}_{knee,i}\right)-D_{knee}{\overset{∙}{\theta }}_{knee,i}, \end{array}$$

(3) $$\begin{array}{r} \tau_{ankle,i}=-K_{ankle}\left(\theta_{ankle,i}-{\bar{\theta }}_{ankle,i}\right)-D_{ankle}{\overset{∙}{\theta }}_{ankle,i}, \end{array}$$

(4) $$\begin{array}{r} \tau_{trunk}=-K_{trunk}\left(\theta_{trunk}-{\bar{\theta }}_{trunk}\right)-D_{trunk}{\overset{∙}{\theta }}_{trunk}, \end{array}$$

where θ_*y, i*_ and ${\bar{\theta }}_{y,i}$ represent the actual and target angles, respectively, for Joint *y* in the *ith* leg, and *K*_*y*_ and *D*_*y*_ represent the proportional and derivative gains of the PD controller for Joint *y*, respectively. The hip, knee, and ankle joint controllers use the Tegotae function to modulate the target angles ${\bar{\theta }}_{y,i}$ or proportional gains *K*_*y*_ for adaptive walking. The parameters for PD gains are shown in Supplementary Tables 1, 2. The remaining section describes the Tegotae function and concept of Tegotae-based control, including a comprehensive explanation of each controller.

#### 2.2.1. Tegotae Functions

As explained in section 1, Tegotae is a Japanese concept centred around the extent to which a generated action matches a perceived reaction. In robotics, it is the consistency between the intended motor command from a controller and received sensory information based on the motion generated by the controller. Thus, quantification of the Tegotae concept yielded a Tegotae function that can be described as the product of the (i) intended motor command of a controller *f*(*x*), where *x* denotes the control variable, and (ii) resulting sensory information *g*(*S*) obtained in the form of sensor values, *S*, as follows:

(5) $$\begin{array}{r} T\left(x,S\right)=f\left(x\right)g\left(S\right). \end{array}$$

The Tegotae function was created such that the positive/negative values output by the function indicate consistency/inconsistency between the intended motor command and resulting sensory information.

#### 2.2.2. Tegotae-Based Control

Using the Tegotae function *T*(*x, S*), we can modulate the localised control variable *x* as follows:

(6) $$\begin{array}{r} ẋ=h\left(x\right)+\frac{\partial T\left(x,S\right)}{\partial x}, \end{array}$$

where the first term on the right represents the intrinsic dynamics of the localised controller, and second term represents the Tegotae-based localised sensory feedback for the control variable *x*. Using the sensory feedback determined by the partial differential form of the Tegotae function, the controller can modulate its control variable *x* such that it maximises the Tegotae-based consistency with the expectation. Thus, we can design a systematic control scheme for many components by creating Tegotae functions for each controller. We next describe the localised hip, knee, and ankle joint controllers with Tegotae function-based designs.

### 2.3. Design of Joint Controllers

#### 2.3.1. Hip Control

**Figure 2:**
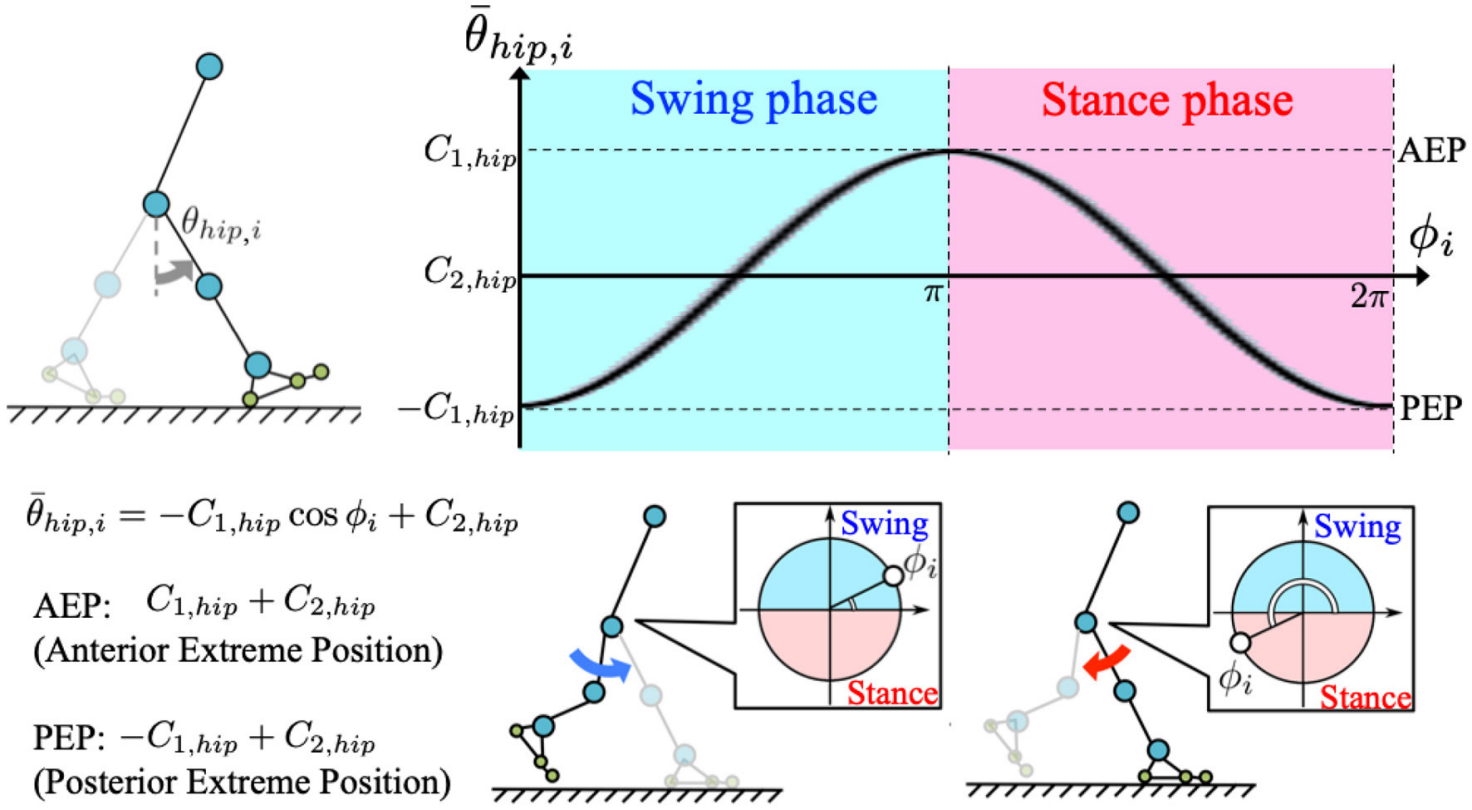
Generation of target angle in hip joint control: we incorporated a phase oscillator as a component of the CPG-based model to generate target angle for the corresponding hip actuator. The target angle is described by ${\bar{\theta }}_{hip,i}=-C_{1,hip}\cos\phi_{i}+C_{2,hip}$, where *C*_1, *hip*_, and *C*_2, *hip*_ are amplitude and offset angles of the hip target angle, respectively. According to the equation, posterior and anterior extreme positions (PEP and AEP, respectively) of the target angle result in −*C*_1, *hip*_+*C*_2, *hip*_(ϕ_*i*_ = 0, 2π) and *C*_1, *hip*_+*C*_2, *hip*_(ϕ_*i*_ = π), respectively. Therefore, legs are controlled to be in the swing phase for 0 ≤ ϕ_*i*_ < π, and in the stance phase for π ≤ ϕ_*i*_ < 2π.

The role of the hip joints in human gait is to generate rhythmic forward and backward leg-swinging movements (Perry and Burnfield, 2010). To enable such rhythmic movements, we incorporated phase oscillators as a component of the CPG-based model to generate the target angle for the hip actuators (Equation 1) as follows:

(7) $$\begin{array}{r} {\bar{\theta }}_{hip,i}=-C_{1,hip}\cos\phi_{i}+C_{2,hip}, \end{array}$$

where *C*_1, *hip*_ and *C*_2, *hip*_ [rad], respectively, represent the amplitude and offset components of the hip target angle. When implementing the oscillator phases, legs are controlled to remain in the swing phase for 0 ≤ ϕ_*i*_ < π, and stance phase for π ≤ ϕ_*i*_ < 2π (Figure 2).

The dynamics of the phase oscillators with the localised Tegotae function-based sensory feedback can be described as follows:

(8) $$\begin{array}{r} {\overset{∙}{\phi }}_{i}=\omega +\frac{\partial T_{hip,i}\left(\phi_{i},N\right)}{\partial \phi_{i}}, \end{array}$$

where ω [rad/s] represents the intrinsic angular velocity of the oscillators. The Tegotae function for hip control has been defined as follows:

(9) $$\begin{array}{r} T_{hip,i}\left(\phi_{i},N\right)=\sigma_{hip,1}\left\{N_{h,i}^{V}\left(-\text{sin}\phi_{i}\right)+\left(N_{m,i}^{V}+N_{t,i}^{V}\right)\left(\text{sin}\phi_{i}\right)\right\}\\ \;+\sigma_{hip,2}\left\{N_{h,j}^{V}\left(\text{sin}\phi_{i}\right)+\left(N_{m,j}^{V}+N_{t,j}^{V}\right)\left(-\text{sin}\phi_{i}\right)\right\}, \end{array}$$

where σ_*hip*, 1_ and σ_*hip*, 2_ [rad/Ns] represent the feedback gains. The suffixes *i* and *j* denote the corresponding leg and other leg, respectively.

**Figure 3:**
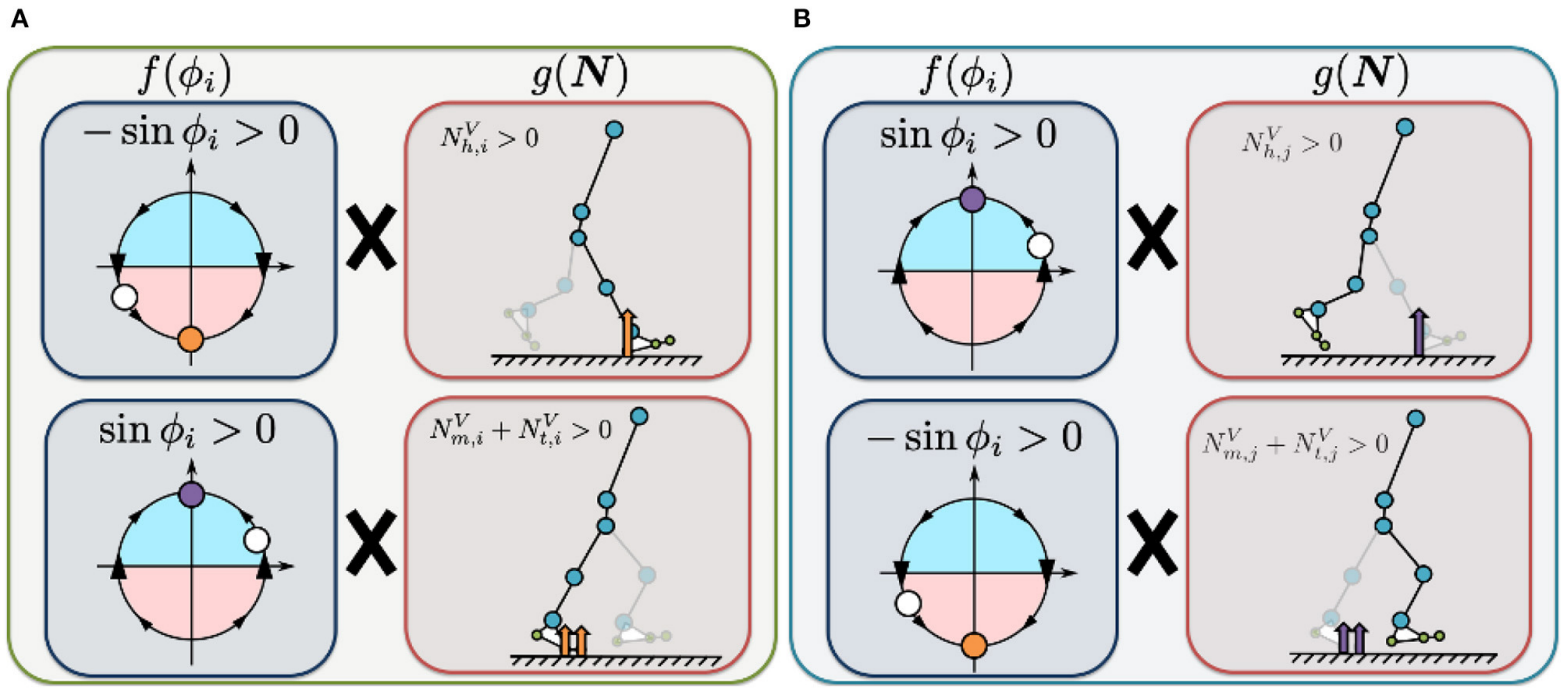
Illustrated definition of the Tegotae function for the hip controller. For rhythmic movements, we incorporated phase oscillators as a component of the CPG-based model to generate the target angle for the hip actuators (Equation 8). **(A)** Tegotae function for corresponding-leg sensory information. The value of $N_{h,i}^{V}\left(-\text{sin}\phi_{i}\right)$ is positive when the heel sensor on the corresponding leg detects a large vertical GRF ($N_{h,i}^{V}>0$) with the oscillator in the stance phase (π ≤ ϕ_*i*_ < 2π). In contrast, the value of ($N_{m,i}^{V}+N_{t,i}^{V}$) (sinϕ_*i*_) is positive when the metatarsal and toe sensors on the corresponding leg detect a large vertical GRF ($N_{m,i}^{V}+N_{t,i}^{V}>0$) with oscillator in the swing phase (0 ≤ ϕ_*i*_ < π). **(B)** Tegotae function for opposite-leg sensory information. The value of $N_{h,j}^{V}(\text{sin}\phi_{i}$) is positive when the heel sensor on the opposite leg detects a large vertical GRF ($N_{h,j}^{V}>0$), with the oscillator in the swing phase (0 ≤ ϕ_*i*_ < π). In contrast, the value of ($N_{m,j}^{V}+N_{t,j}^{V}$) (−sinϕ_*i*_) is positive when the metatarsal and toe sensors on the opposite leg detect a large vertical GRF ($N_{m,j}^{V}+N_{t,j}^{V}>0$) with the oscillator in the stance phase (π ≤ ϕ_*i*_ < 2π). The white circles represent the corresponding oscillator phase ϕ_*i*_. The orange and purple circles represent stable equilibrium points of Tegotae-based feedback, e.g., $\frac{\partial \left(\text{sin}\phi_{i}\right)}{\partial \phi_{i}}=\cos\phi_{i}=0$ for the top of **(A)** (the first term in Equation 9).

The first term on the right describes how the Tegotae function is applied in the case of sensory information for the corresponding leg (Figure 3A). The value of $N_{h,i}^{V}\left(-\text{sin}\phi_{i}\right)$ is positive when the heel sensor on the corresponding leg detects a large vertical GRF ($N_{h,i}^{V}>0$) with the oscillator in the stance phase (π ≤ ϕ_*i*_ < 2π). Increasing this Tegotae term allows the leg to remain in the stance phase as it supports the body ($N_{h,i}^{V}>0$). In contrast, the value of ($N_{m,i}^{V}+N_{t,i}^{V}$) (sinϕ_*i*_) is positive when the metatarsal and toe sensors on the corresponding leg detect a large vertical GRF ($N_{m,i}^{V}+N_{t,i}^{V}>0$) with the oscillator in the swing phase (0 ≤ ϕ_*i*_ < π). In this case, increasing the Tegotae term results in the leg transitioning from the stance to swing phase ($N_{m,i}^{V}+N_{t,i}^{V}>0$), propelling the body forward.

The second term describes how the Tegotae function is applied in the case of sensory information for the opposite leg (Figure 3B). The value of $N_{h,j}^{V}(\text{sin}\phi_{i}$) is positive when the heel sensor on the opposite leg detects a large vertical GRF ($N_{h,j}^{V}>0$), with the oscillator in the swing phase (0 ≤ ϕ_*i*_ < π). Increasing this Tegotae term allows the corresponding leg to remain in the swing phase as the opposite leg supports the body ($N_{h,j}^{V}>0$); this support allows the corresponding leg to complete the swing phase successfully. In contrast, the value of ($N_{m,j}^{V}+N_{t,j}^{V}$) (−sinϕ_*i*_) is positive when the metatarsal and toe sensors on the opposite leg detect a large vertical GRF ($N_{m,j}^{V}+N_{t,j}^{V}>0$) with the oscillator in the stance phase (π ≤ ϕ_*i*_ < 2π). Under these conditions, an increase in the Tegotae term results in the corresponding leg initiating a smooth transition from the swing to stance phase ($N_{m,j}^{V}+N_{t,j}^{V}>0$). Here, we do not use any neural synaptic connections between the hip oscillators; previous studies achieved the desired rhythmic walking motion by manually setting the neural synaptic connectivity parameters in advance (e.g., Taga et al., 1991; Nassour et al., 2014). Implementation of the Tegotae-based localised feedback scheme described by Equation (8) allows the hip controllers to achieve interlimb coordination in the absence of any neural communication between oscillators.

#### 2.3.2. Knee Control

The roles of a knee joint in human gait (Perry and Burnfield, 2010) are as follows: (1) support the body by increasing its stiffness in the stance phase (2) increase the effective flexion by reducing its stiffness in the swing phase. Thus, we established the control variable χ_*i*_, representing the control command that increases/decreases the knee joint stiffness. To implement this stiffness control mechanism, we use χ_*i*_ to adjust the gain *K*_*knee, i*_ in the knee controllers, as follows:

(10) $$\begin{array}{r} \tau_{knee,i}=-K_{knee,i}\left(\theta_{knee,i}-{\bar{\theta }}_{knee,i}\right)-D_{knee}{\overset{∙}{\theta }}_{knee,i}, \end{array}$$

(11) $$\begin{array}{r} K_{knee,i}=\max\left[C_{1,knee}\tanh\chi_{i},0\right]+C_{2,knee}, \end{array}$$

where *C*_1, *knee*_ and *C*_2, *knee*_ [Nm/rad] represent the variable range and offset value of the gain *K*_*knee, i*_, respectively. We used tanh function to model continuous on/off-like function (scaled from −1.0 to 1.0) according to the control variable χ_*i*_. In Equation (2), the target angle ${\bar{\theta }}_{knee}$ for the knee controllers was set to 0 [rad]; this angle indicates the degree of knee extension and determines whether the stiffness should be increased/decreased to extend/flex the knee joint.

**Figure 4:**
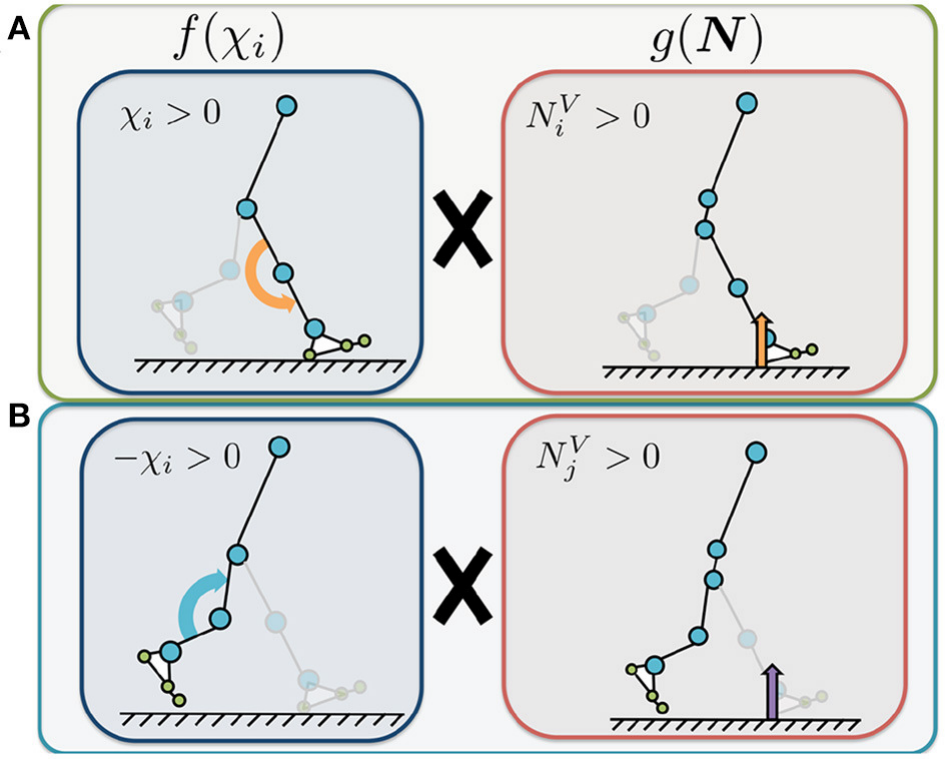
Illustrated definition of the Tegotae function for the knee controller. The control variable χ_*i*_ represents the control command that increases or decreases the knee joint stiffness (Equation 9). We use χ_*i*_ to adjust the P-gain *K*_*knee, i*_ in the knee controllers for the implementation of stiffness control mechanism, in Equation (10). $N_{i}^{V}$ and $N_{j}^{V}$ represent the sums of the vertical force sensor values corresponding to the heel, metatarsal, and toe joints of the corresponding (*i*) and opposite (*j*) legs, respectively. **(A)** Tegotae function for corresponding-leg sensory information. The value of $N_{i}^{V}\chi_{i}$ is positive when the foot sensors on the corresponding leg detect a large vertical GRF ($N_{i}^{V}>0$) and the control command for the knee is to increase the stiffness (i.e., χ_*i*_ > 0). **(B)** Tegotae function for opposite-leg sensory information. The value of $N_{j}^{V}\left(-\chi_{i}\right)$ is positive when the foot sensors on the opposite leg detect a large vertical GRF ($N_{j}^{V}>0$) and the control command for the knee is to decrease the stiffness (i.e., χ_*i*_ < 0).

The dynamics of the control variable χ_*i*_ for the localised Tegotae function-based sensory feedback scheme can be described as follows:

(12) $$\begin{array}{r} {\overset{∙}{\chi }}_{i}=-c_{knee}\chi_{i}+\frac{\partial T_{knee,i}\left(\chi_{i},N\right)}{\partial \chi_{i}}, \end{array}$$

where *c*_*knee*_ represents the parameter related to its response time for the first-order dynamical model of the knee controller. The reason for choosing a first-order model was its simplicity (only one parameter *c*_*knee*_) and non-rhythmic behaviour, meaning that it stays at a equilibrium point (χ_*i*_ = 0) without feedback. The Tegotae function for knee control is defined as follows:

(13) $$\begin{array}{r} T_{knee,i}\left(\chi_{i},N\right)=\sigma_{knee,1}N_{i}^{V}\chi_{i}+\sigma_{knee,2}N_{j}^{V}\left(-\chi_{i}\right), \end{array}$$

(14) $$\begin{array}{r} N_{i}^{V}=N_{h,i}^{V}+N_{m,i}^{V}+N_{t,i}^{V}, \end{array}$$

(15) $$\begin{array}{r} N_{j}^{V}=N_{h,j}^{V}+N_{m,j}^{V}+N_{t,j}^{V}, \end{array}$$

where $N_{i}^{V}$ and $N_{j}^{V}$ [N] represent the sums of the vertical force sensor values corresponding to the heel, metatarsal, and toe joints of the corresponding and opposite legs, respectively. The parameters σ_*knee*, 1_ and σ_*knee*, 2_ [1/N] represent the feedback gains. The first term on the right represents the Tegotae function for the corresponding leg (Figure 4A). The value of $N_{i}^{V}\chi_{i}$ is positive when the foot sensors on the corresponding leg detect a large vertical GRF ($N_{i}^{V}>0$) and the control command for the knee is to increase the stiffness (i.e., χ_*i*_ > 0). Increasing this Tegotae term causes the knee stiffness to remain high to ensure that the body is supported ($N_{i}^{V}>0$). The second term represents the Tegotae function for the opposite leg (Figure 4B). The value of $N_{j}^{V}\left(-\chi_{i}\right)$ is positive when the foot sensors on the opposite leg detect a large vertical GRF ($N_{j}^{V}>0$) and the control command for the knee is to decrease the stiffness (i.e., χ_*i*_ < 0). Increasing this Tegotae term ensures that the knee stiffness remains low to allow the knee to bend during the swing phase as the opposite leg supports the body ($N_{j}^{V}>0$); this state allows the corresponding leg to swing forward in the swing phase.

#### 2.3.3. Ankle Control

The role of an ankle joint in human gait (Perry and Burnfield, 2010) is to generate the propulsive forces necessary for the leg to transition from the stance to swing phase while avoiding a collision between the foot and ground. Therefore, we established the control variable ψ_*i*_ for the ankle controllers, which represents the control command that increases or decreases the target angle of the ankle joints as follows:

(16) $$\begin{array}{r} {\bar{\theta }}_{ankle,i}=C_{1,ankle}\tanh\psi_{i}+C_{2,ankle}, \end{array}$$

where *C*_1, *ankle*_ and *C*_2, *ankle*_ [rad] represent the variable range and offset value of the ankle target angle, respectively. We used tanh function to model continuous on/off-like function (scaled from −1.0 to 1.0) according to the control variable ψ_*i*_. A positive/negative value of ψ_*i*_ represents the plantar/dorsal flexion of an ankle joint.

**Figure 5:**
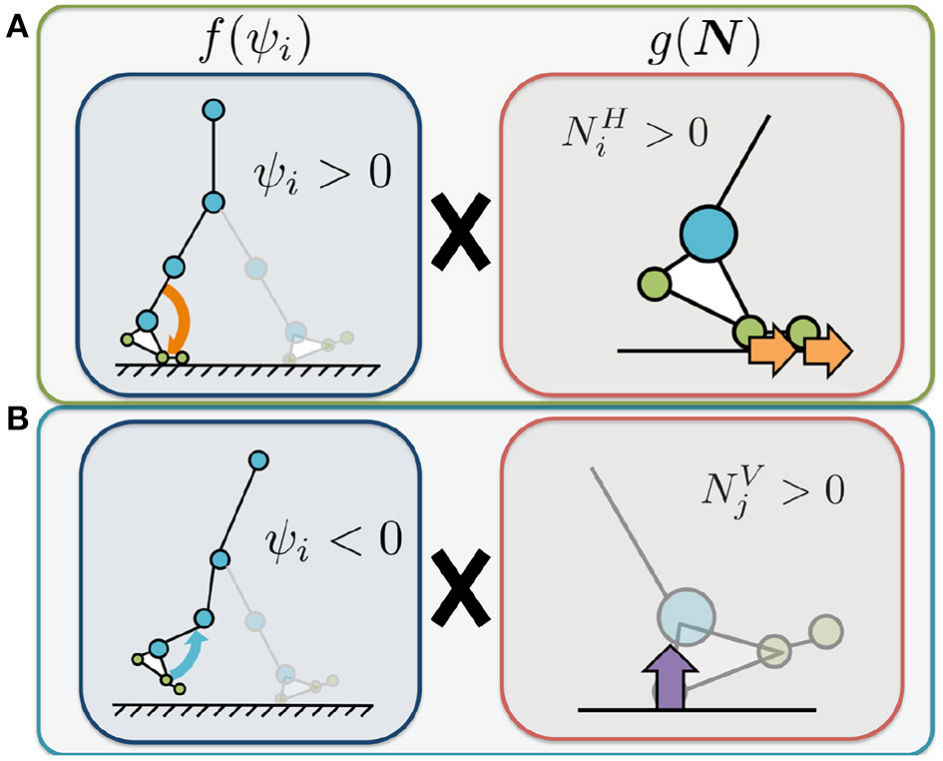
Illustrated definition of the Tegotae function for the ankle controller. The control variable ψ_*i*_ represents the control command that increases or decreases the target angle of the ankle joints. We use ψ_*i*_ to adjust the target angle for the ankle controllers. $N_{i}^{H}$ represents the sum of the horizontal force sensor values corresponding to the heel, metatarsal, and toe joints of the corresponding leg. $N_{j}^{V}$ represents the sum of the vertical force sensor values corresponding to the opposite leg. **(A)** Tegotae function for corresponding-leg sensory information. The value of $N_{i}^{H}\psi_{i}$ is positive when the foot sensors on the corresponding leg detect a large horizontal GRF ($N_{i}^{H}>0$) and the command for the ankle is plantar flexion (i.e., ψ_*i*_ > 0). **(B)** Tegotae function for opposite-leg sensory information. The value of $N_{j}^{V}\left(-\psi_{i}\right)$ is positive when the foot sensors on the opposite leg detect a large vertical GRF ($N_{j}^{V}>0$) and the command for the ankle is dorsal flexion (ψ_*i*_ < 0).

The dynamics of the control variable ψ_*i*_ for the localised Tegotae function-based sensory feedback method can be described as follows:

(17) $$\begin{array}{r} {\overset{∙}{\psi }}_{i}=-c_{ankle}\psi_{i}+\frac{\partial T_{ankle,i}\left(\psi_{i},N\right)}{\partial \psi_{i}}, \end{array}$$

where *c*_*ankle*_ represents the parameter related to its response time for the first-order dynamical model of the ankle controller. The Tegotae function for ankle control is defined as follows:

(18) $$\begin{array}{r} T_{ankle,i}\left(\psi_{i},N\right)=\sigma_{ankle,1}N_{i}^{H}\psi_{i}+\sigma_{ankle,2}N_{j}^{V}\left(-\psi_{i}\right), \end{array}$$

(19) $$\begin{array}{r} N_{i}^{H}=N_{h,i}^{H}+N_{m,i}^{H}+N_{t,i}^{H}, \end{array}$$

(20) $$\begin{array}{r} N_{j}^{V}=N_{h,j}^{V}+N_{m,j}^{V}+N_{t,j}^{V}, \end{array}$$

where $N_{i}^{H}$ [N] represents the sum of the horizontal force sensor values corresponding to the heel, metatarsal, and toe joints of the corresponding leg; Similarly, $N_{j}^{V}$ [N] represents the sum of the vertical force sensor values corresponding to the opposite leg; The parameters σ_*ankle*, 1_ and σ_*ankle*, 2_ [1/N] represent the feedback gains. The first term on the right represents the Tegotae function for the corresponding leg (Figure 5A). The value of $N_{i}^{H}\psi_{i}$ is positive when the foot sensors on the corresponding leg detect a large horizontal GRF ($N_{i}^{H}>0$) and the command for the ankle is plantar flexion (i.e., ψ_*i*_ > 0). Increasing this Tegotae term results in stronger plantar flexion at the end of the stance phase ($N_{i}^{H}>0$), thus generating a larger propulsive force. The second term represents the Tegotae function for the opposite leg (Figure 5B). The value of $N_{j}^{V}\left(-\psi_{i}\right)$ is positive when the foot sensors on the opposite leg detect a large vertical GRF ($N_{j}^{V}>0$) and the command for the ankle is dorsal flexion (ψ_*i*_ < 0). Increasing this Tegotae term allows the ankle joint controller to effectively generate the dorsal flexion strength necessary for ground clearance during the swing phase as the opposite leg supports the body ($N_{j}^{V}>0$).

To reiterate, the dynamics of each of the joint controller designs can be described as follows:

(21) $$\begin{array}{r} {\overset{∙}{\phi }}_{i}=\omega +\sigma_{hip,1}\left(-N_{h,i}^{V}+N_{m,i}^{V}+N_{t,i}^{V}\right)\cos\phi_{i}\\ +\sigma_{hip,2}\left(N_{h,j}^{V}-N_{m,j}^{V}-N_{t,j}^{V}\right)\cos\phi_{i}, \end{array}$$

(22) $$\begin{array}{r} {\overset{∙}{\chi }}_{i}=-c_{knee}\chi_{i}+\sigma_{knee,1}\left(N_{h,i}^{V}+N_{m,i}^{V}+N_{t,i}^{V}\right)\\ -\sigma_{knee,2}\left(N_{h,j}^{V}+N_{m,j}^{V}+N_{t,j}^{V}\right), \end{array}$$

(23) $$\begin{array}{r} {\overset{∙}{\psi }}_{i}=-c_{ankle}\psi_{i}+\sigma_{ankle,1}\left(N_{h,i}^{H}+N_{m,i}^{H}+N_{t,i}^{H}\right)\\ -\sigma_{ankle,2}\left(N_{h,j}^{V}+N_{m,j}^{V}+N_{t,j}^{V}\right), \end{array}$$

The advantage of implementing the Tegotae functions is that it allows us to systematically design controllers for various joint types for the robot to perform the target movements. Furthermore, we expect that the sensory information (i.e., GRFs) utilised by the Tegotae-based hip, knee, and ankle joint controllers will enable spontaneous and adaptive inter- and intra-limb coordination.

#### 2.3.4. Postural Control for the Trunk

**Figure 6:**
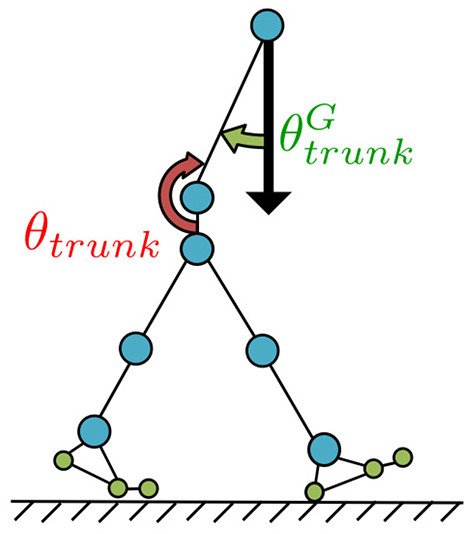
Postural control for the trunk joint. The trunk joint was designed to be controlled such that the angle $\theta_{trunk}^{G}$ (angle between the torso link and direction of gravitational acceleration), which is detected by the vestibular sensor, can be accurately represented by the fixed angle α for preventing destabilising forward and backward upper-body movement.

To prevent destabilising forward and backward upper-body movement, the trunk joint was designed to be controlled such that the angle $\theta_{trunk}^{G}$ between the torso link and direction of gravitational acceleration, which is detected by the vestibular sensor, can be accurately represented by the fixed angle α (Figure 6), as described by the following equations:

(24) $$\begin{array}{r} \tau_{trunk}=-K_{trunk}\left(\theta_{trunk}-{\bar{\theta }}_{trunk}\right)-D_{trunk}{\overset{∙}{\theta }}_{trunk}, \end{array}$$

(25) $$\begin{array}{r} {\bar{\theta }}_{trunk}=\alpha -\theta_{trunk}^{G}, \end{array}$$

where ${\bar{\theta }}_{trunk}$ represents the target angle for the PD controller at the trunk joint, and *K*_*trunk*_ and *D*_*trunk*_ are the proportional and derivative gains of the PD controller, respectively.

## 3. Simulation Results

### 3.1. Steady Walking

**Figure 7:**
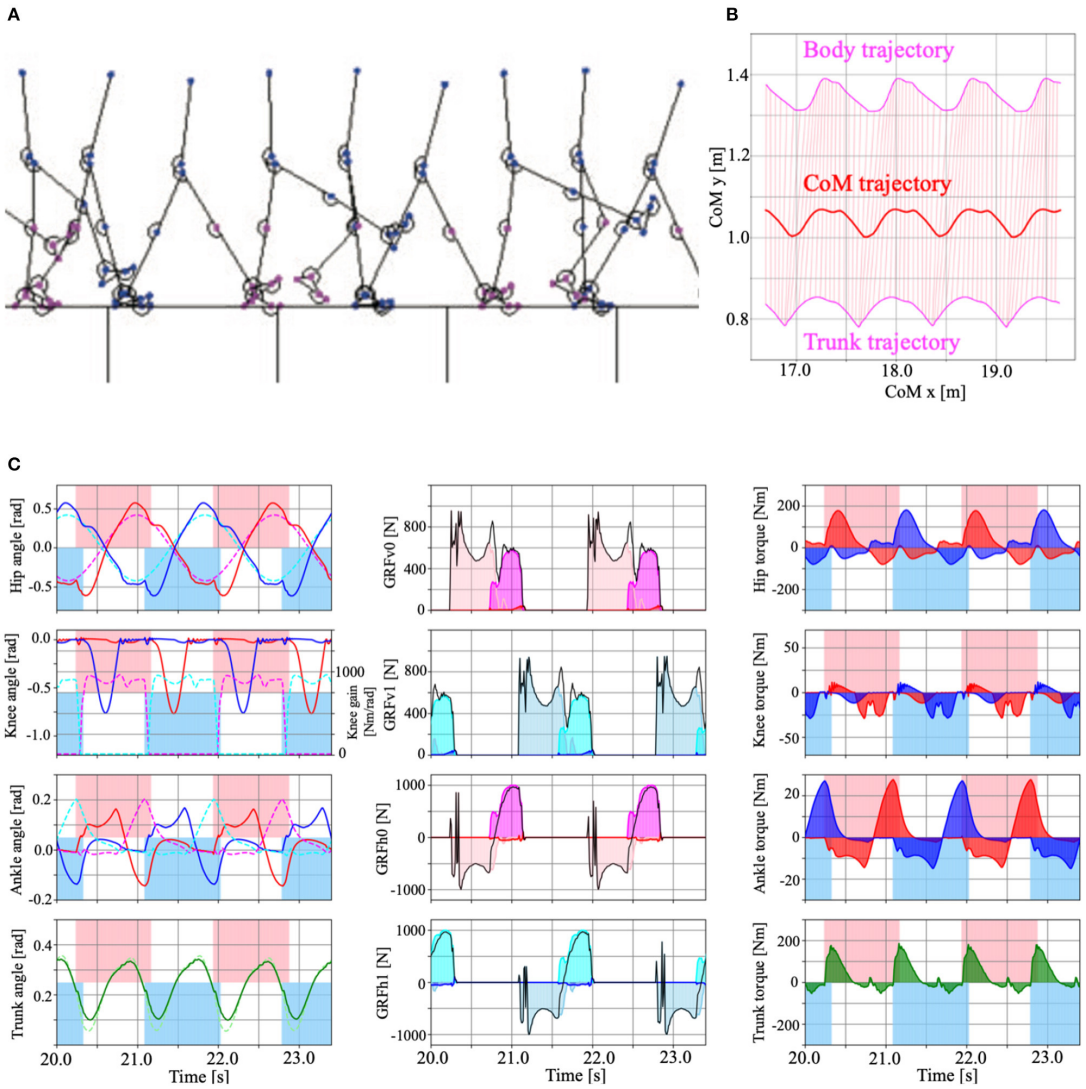
**(A)** Example of the model walking in steady-state (Model 3 with PC, Supplementary Movie 1). **(B)** Body, trunk (magenta), and CoM (Centre of Mass, red) trajectories during the steady walking. Pink lines represent the torso link that connects the body and trunk mass. **(C)** Time series data of the steady walking in Model 3 with PC. Left panels show the hip, knee, ankle, and trunk angle from top to bottom. The red and blue lines show the left and right legs, respectively. The dotted magenta (left leg), cyan (right leg), and light green (trunk) lines represent the target angle of the hip, ankle and trunk, and the knee gain that determined by Tegotae-based control. The centre panels show vertical (top, second) and horizontal (third, bottom) ground reaction forces (GRFs). The pink, magenta, and red coloured regions represent the vertical forces applied to the heel, metatarsals, and toe masses on the left leg. The sky blue, cyan, and blue coloured regions represent the horizontal forces applied to the heel, metatarsals, and toe masses on the right leg. The right panels represent the torque applied to the hip, knee, ankle, and trunk joints by the PD control. The red, blue, and green colour regions represent the left, right legs, and trunk, respectively. For the left and right panels, the pink and sky blue coloured regions represent the stance phase of the left and right legs, determined by the vertical GRFs ($N_{i}^{V}>0$), respectively.

In this section, we present the results of the numerical simulations performed in this study to validate our proposed design scheme. We set the body and control parameters in our bipedal model as shown in Supplementary Table 2 (Supplementary Material). In this study, we derived the control parameters through the trial and error method. Figure 7A is a screenshot of the steady-walking simulation (Supplementary Movie 1), with *Tegotae*-based controls for the hip, knee, and ankle joints and posture control for the trunk joint. Figure 7B shows the body, trunk, and CoM (Center of Mass) trajectories during the steady walking. Figure 7C presents the steady-walking time-series data in Model 3 with PC for the angle of each joint (including trunk angle), target angles of the hip and ankle joints, knee joint gain, vertical and horizontal GRFs, generated torque at each joint, and stance phase duration. By adjusting the target angles of the hip and ankle joints and the knee gain based on Tegotae control, the appropriate timing and magnitude of torques were generated for steady walking. Furthermore, the time-series pattern reproduced in the model was remarkably similar to that of a human steady-state walking time series (Supplementary Figure 3), except for the trunk angle, demonstrating the ability of Tegotae control in extracting the essential aspects of human walking control. We also compared walking speed and Froude numbers with human and other robots in Supplementary Table 3 (Supplementary Material).

### 3.2. Adaptability to Uneven Terrain

**Figure 8:**
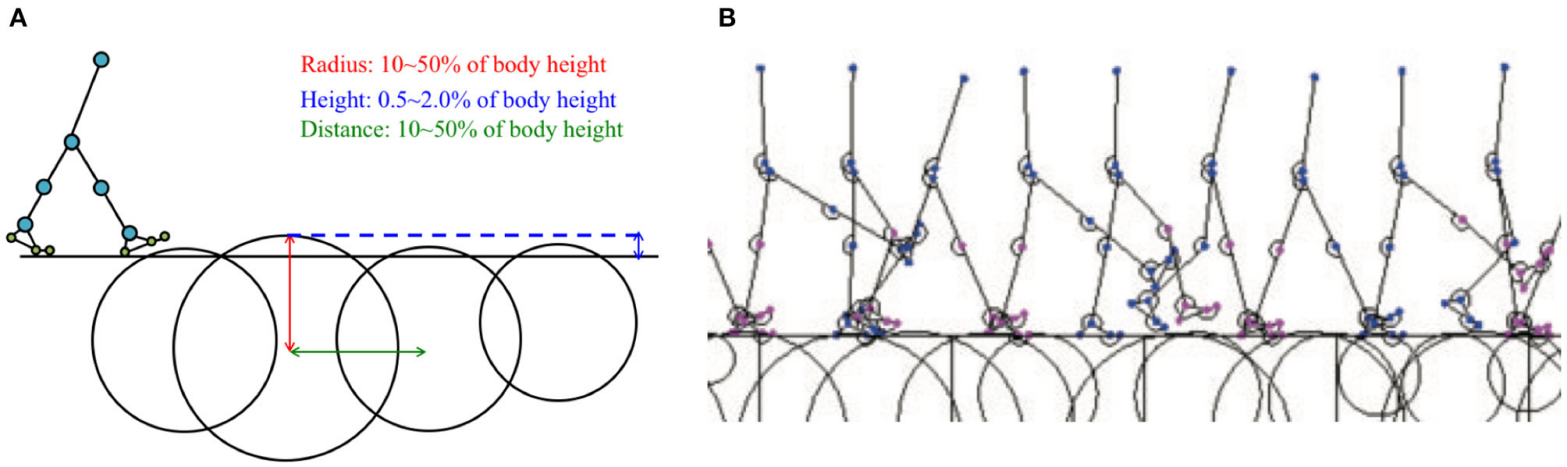
**(A)** Example of simulated environment used to verify model adaptability to uneven terrain. **(B)** Example of stick model completing five walking periods on uneven terrain (Model 3 with PC, Supplementary Movie 1).

Here, we present examples of the simulated results that were subsequently analysed to evaluate the adaptability of the proposed model to environmental changes. To verify the adaptability, we modelled uneven terrains in the simulations by embedding circle obstacles into the ground, as shown in Figure 8A. Here, we again used *Tegotae*-based controls for the hip, knee, and ankle joints and posture control for the trunk joint. The radii of the circle obstacles and distances between the obstacles were randomly selected from values within the range of 10–50% of the body height of the model. The height of each obstacle was also randomly selected from values within the range of 0.5–2.0% of the body height. Figure 8B shows a screenshot of the uneven-terrain simulation. The results obtained via this simulation indicate that the Tegotae-based control scheme can be successfully implemented to allow a bipedal walking robot to adapt to environmental changes.

To investigate the extent of the contributions of the joint controllers to the observed adaptability, we tested the adaptability of the walking model under the following conditions:

- Model 1: Tegotae-based knee controller (σ_*knee, k*_≠0, σ_*hip, k*_ = σ_*ankle, k*_ = 0)
- Model 2: Tegotae-based knee and ankle controllers (σ_*knee, k*_, σ_*ankle, k*_≠0, σ_*hip, k*_ = 0)
- Model 3: Tegotae-based hip, knee, and ankle controllers (σ_*hip, k*_, σ_*knee, k*_, σ_*ankle, k*_≠0)

**Figure 9:**
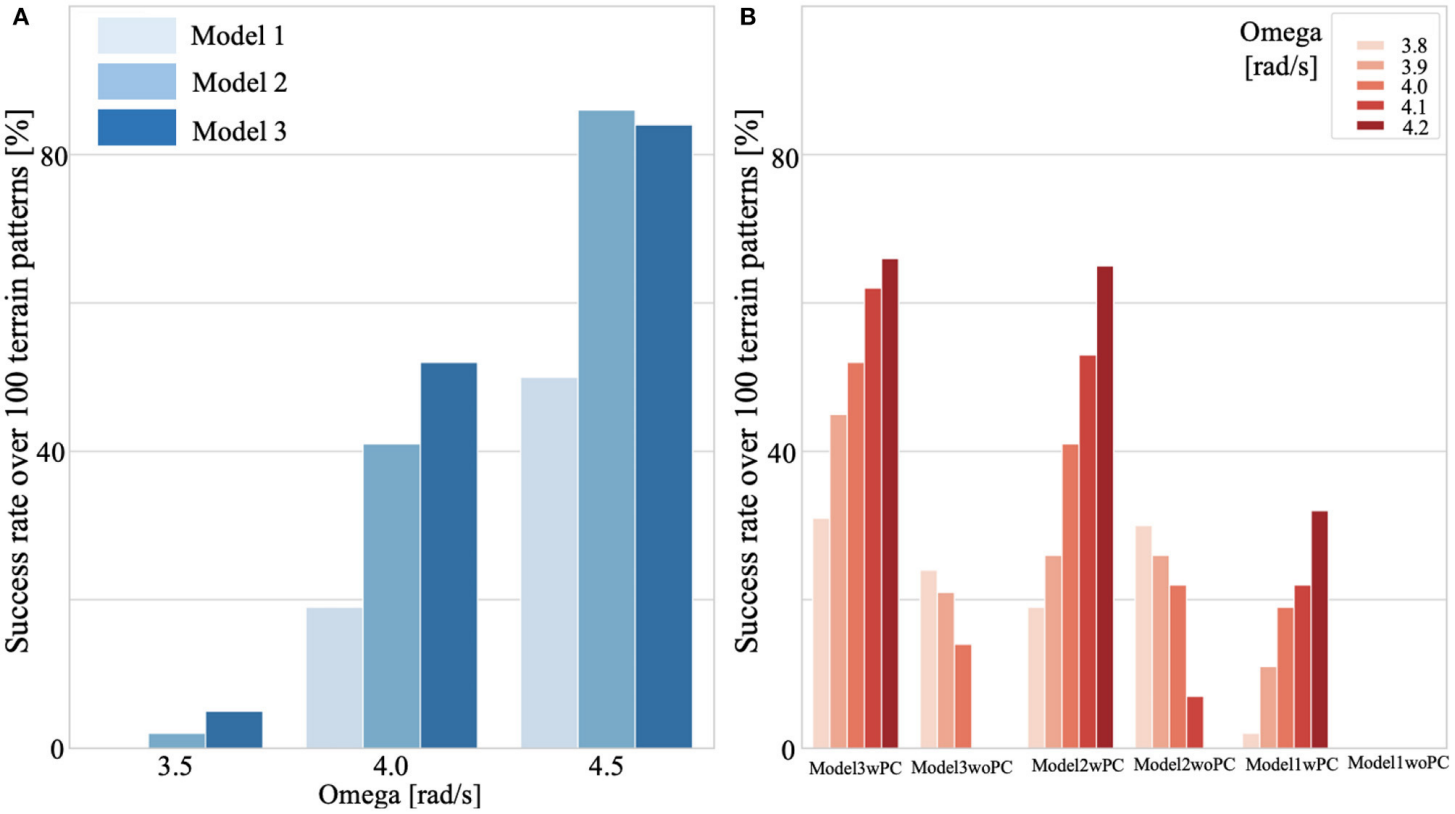
Adaptability on uneven terrain. We simulated test environments that consisted of 10 m of flat ground, followed by 20 m of uneven terrain, and another 10 m of flat ground (Supplementary Movie 1). We verified the walking performance for 100 randomly generated uneven-terrain patterns for each model (1,2,3) [with/without posture control (PC)], and for each control parameter. We judged the success case as the condition in which the bipedal model successfully walked 40 m ground. **(A)** Comparison of success rates for continuous walking on 100 uneven terrain patterns. All models included PC. **(B)** Effects of PC on adaptability. Model 3 with PC shows high environmental adaptability to a wide range of ω. Note that the same terrain patterns were applied in all cases.

All the models include posture control. We simulated a test environment that consisted of 10 m of flat ground, followed by 20 m of uneven terrain (refer to the above-described method), and another 10 m of flat ground (Supplementary Movie 1). We verified the walking performance for 100 randomly generated uneven-terrain patterns. Figure 9A presents a comparison of the success rates of these three models for different oscillator angular velocities. We judged the success case as the condition in which the bipedal model successfully walked 40 m ground in total. These results indicated that Model 3 showed higher adaptability in a wide range of ω from low speed to high speed, whereas Model 2 and Model 3 showed almost the same adaptability on the uneven terrain in high speed walking (ω = 4.5 rad/s) (Figures 9A,B). However, for ω exceeding 4.5, the walking speed decreases along with the increase of ω. One possible reason is that the model used in this study cannot reproduce the running motion.

### 3.3. Effects of Postural Control

To investigate the extent of the contribution of the posture controller to the observed adaptability, we conducted tests to evaluate the adaptability of the walking model with and without postural control explained in section 2.3.4. For the condition without postural control, we set the target angle of the trunk to be ${\bar{\theta }}_{trunk}=\alpha$. Figure 9B shows the effects of postural control on the adaptability of Models 1, 2, and 3. The results shown in this plot suggest that the posture controller implemented in this study can improve the adaptability of any bipedal walking model. Note that, under some of the simulated no-postural-control conditions (Supplementary Movie 2), particularly for Models 1 and 2, steady walking could not be achieved.

### 3.4. Effect of Control Parameters on Walking

Here, we verified the effect of the control parameters on walking performance (adaptability to environmental changes) when using the Tegotae-based control.

**Figure 10:**
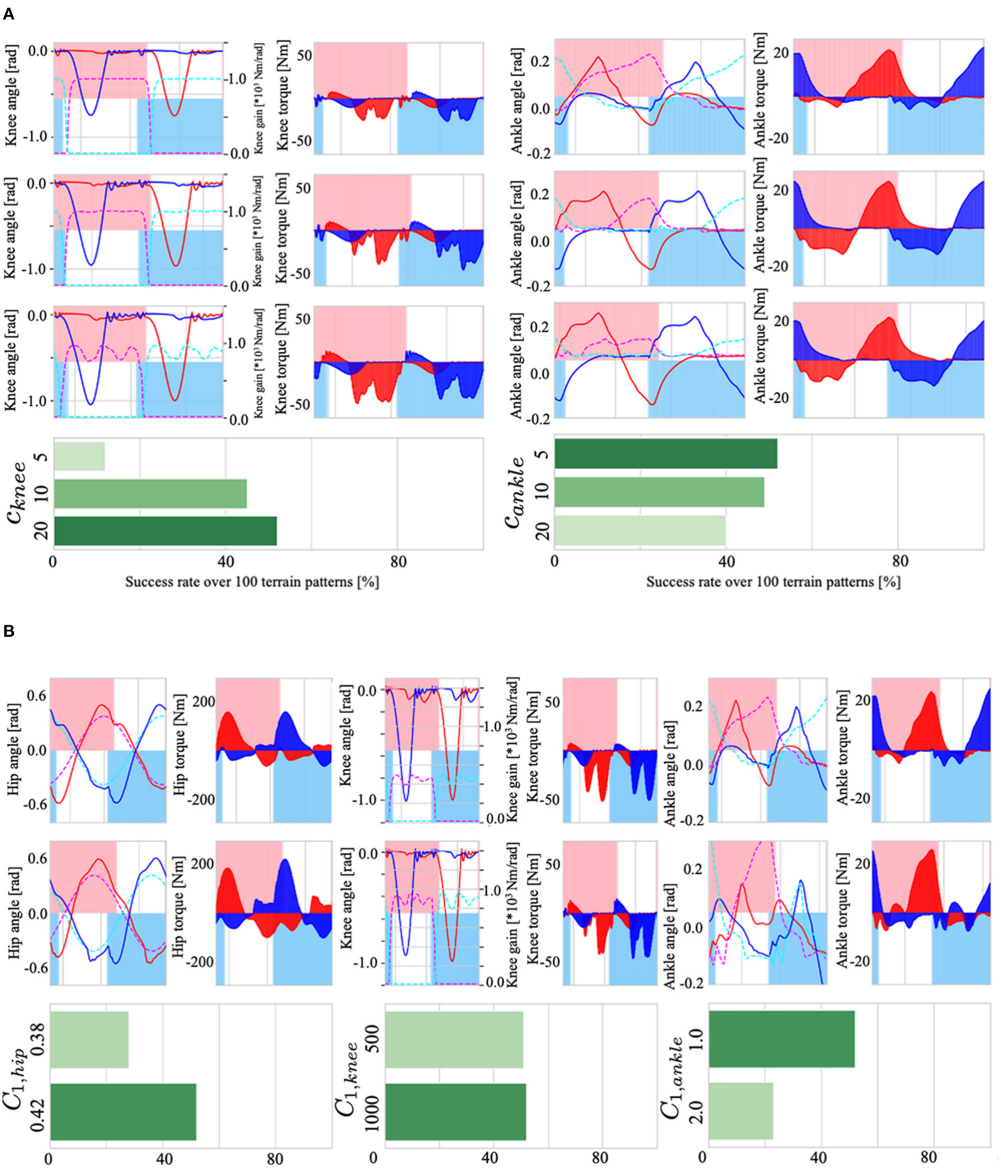
**(A)**: (Left) the effect of *c*_*knee*_ on the knee gain *K*_*knee, i*_, torque τ_*knee, i*_, and walking adaptability. (Right) the effect of *c*_*ankle*_ on the ankle target angle ${\bar{\theta }}_{ankle,i}$, torque τ_*ankle, i*_, and adaptability. **(B)**: (Left) the effect of *C*_1, *hip*_ on the hip joint angle θ_*hip, i*_, torque τ_*hip, i*_, and adaptability. (Center) the effect of *C*_1, *knee*_ on knee gain, torque τ_*knee, i*_, and adaptability. (Right) the effect of *C*_1, *ankle*_ on ankle joint angle θ_*ankle, i*_, torque τ_*ankle, i*_, and adaptability.

First, to verify the effect of the controller dynamics on walking, we tested *c*_*knee*_ and *c*_*ankle*_, which define the dynamics of the knee (Equation 12) and ankle control variables (Equation 17). Figure 10A shows the effect of *c*_*knee*_ on the knee gain *K*_*knee, i*_ and walking adaptability (left), and the effect of *c*_*ankle*_ on the ankle target angle and adaptability (right). The results showed that (i) when *c*_*knee*_ was 20 (reaction time is fast), the Tegotae feedback (the 2nd term of Equation 12) weakened relative to the first term, the gain *K*_*knee, i*_ was fastly modified toward 0 via −*c*_*knee*_χ_*i*_, which improves gait adaptability. When *c*_*knee*_ was small (*c*_*knee*_=5), the effect of feedback was large and the gain *K*_*knee*_ was almost constant in higher value. (ii) When *c*_*ankle*_ was 5 (reaction time is slow), the target angle of the ankle joint changes significantly due to the effect of the Tegotae feedback term (the 2nd term in Equation 17), thus generating sufficient ankle joint torque and improving walking adaptability. When the *c*_*ankle*_ was large, the change in the target angle of the ankle joint was small, resulting in in-sufficient ankle joint torque. In sum, the first order equations exhibit non-rhythmic behaviour, where the control variable stays at an equilibrium point without feedback: the parameters *c*_*knee*_ and *c*_*ankle*_ determine the strength of staying at the equilibrium point (χ_*i*_ = 0, ψ_*i*_ = 0).

Next, we examined the effects of *C*_1, *hip*_, *C*_1, *knee*_, and *C*_1, *ankle*_, the parameters that set amplitude in Equations (7), (11), and (16), which determine the target angle ${\bar{\theta }}_{hip,i},{\bar{\theta }}_{ankle,i}$ and gain *K*_*knee, i*_ of the PD control. The results are shown in Figure 10B; (i) Because *C*_1, *hip*_ is a parameter that determines the amplitude of the target angle ${\bar{\theta }}_{hip,i}$ of the hip joint, setting *C*_1, *hip*_ to a large value increased the amplitude of the hip joint angle θ_*hip, i*_ (Figure 10B, lower left), resulting in an increase in gait stability; (ii) *C*_1, *knee*_ is a parameter that sets the maximum value of the knee P-gain *K*_*knee, i*_ (middle of Figure 10B); changes in the upper limit of the P-gain *K*_*knee, i*_ resulted in an increase in the small oscillations of the knee joint, but there was no significant difference in adaptability; and (iii) *C*_1, *ankle*_ is a parameter that determines the maximum amplitude of the target angle ${\bar{\theta }}_{ankle,i}$ of the ankle joint. Therefore, increasing this parameter increased the range of changes in the ankle joint θ_*ankle, i*_, but had a negative effect on walking adaptability. The reason for this may be that the larger the ankle joint change, the easier it is to trip during walking, leading to falls.

### 3.5. Stability Analysis

**Figure 11:**
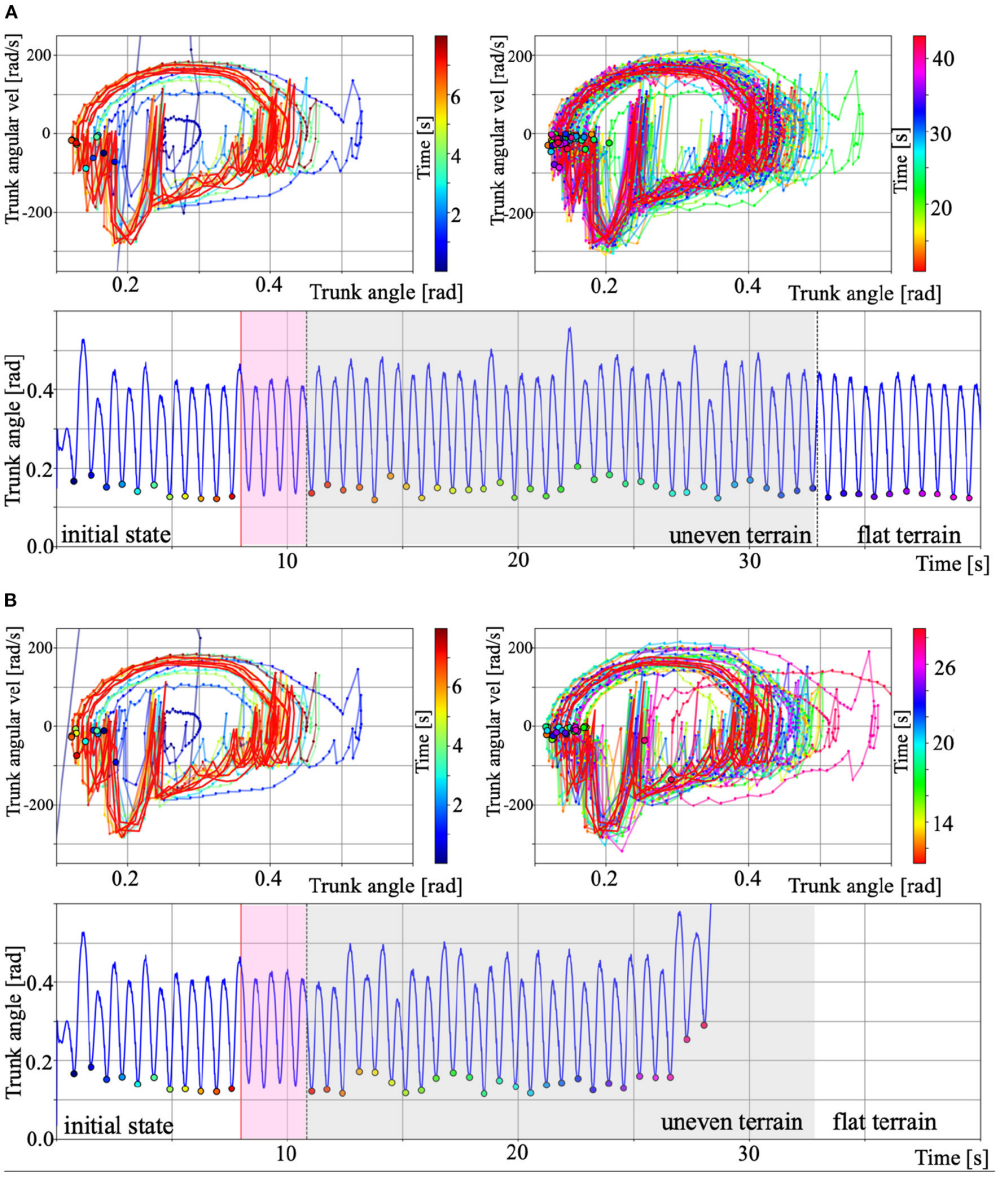
Phase diagram of the trunk angle θ_*trunk*_ and angular velocity ${\overset{∙}{\theta }}_{trunk}$ for the results of section 3.2. **(A)** Gait over uneven terrain. **(B)** Gait that could not move over uneven terrain. For A and B, the upper left and right panels show the period from initial to steady state and during uneven terrain walking. The colour legend for each panel indicate the time [s]. The red trajectories show the limit cycles from 8 s to the beginning of uneven terrain (pink area in the lower panels), which is defined as the steady-state trajectories. The black border points in the phase diagrams indicate the minimum trunk angle (${\overset{∙}{\theta }}_{trunk}=0$) during each walking cycle. We defined this point as the Poincaré section Σ (Nassour et al., 2014). We can then confirm the convergence of the walking to the steady state from the transition process. The lower graphs show the time evolution of the trunk angle θ_*trunk*_. The pink and grey area show the steady-state and period during uneven terrain walking, and the other areas show period during walking on flat terrain.

To numerically verify the stability of the walking motion generated by the Tegotae-based control, we plotted the phase diagram consisting of the trunk angle θ_*trunk*_ and angular velocity ${\overset{∙}{\theta }}_{trunk}$. For testing the adaptability to environmental changes, mentioned in section 3.2 (ω = 4.5 rad/s), we compared the gait that could (Figure 11A) and could not (Figure 11B) move over uneven terrain. The lower graphs of Figures 11A,B show the time evolution of the trunk angle θ_*trunk*_. In both cases (Figures 11A,B), the walking quickly converges from the initial state to the steady state (0 s to around 8 s). The red trajectories in the upper figures show the limit cycle trajectory from 8 s to the beginning of uneven terrain (pink area in the figure below), which is defined as the steady-state trajectories. The black border points in the phase diagrams indicate the minimum trunk angle (${\overset{∙}{\theta }}_{trunk}=0$) during each walking cycle. We defined this state (minimum angle θ_*trunnk*_ and ${\overset{∙}{\theta }}_{trunnk}=0$) as the Poincaré section Σ (Nassour et al., 2014), then, we can confirm the convergence of the walking to the steady state from the transition process (bottom of Figures 11A,B).

The grey areas in the lower figures of Figures 11A,B indicate the period during uneven terrain walking. The upper right figures in Figures 11A,B show the phase diagram during uneven terrain walking. In case A, the trajectory was disturbed by the uneven terrain, but the trajectory was within the basin of attraction of walking, so the biped model can continue to walk on the uneven terrain. In contrast, in case B, the trajectory goes out of the basin of attraction due to uneven terrain, making it impossible to converge to the limit cycle, and the model falls down. This analysis confirms existence of the basin of attraction in walking based on Tegotae control and the destabilisation that caused it to fall over when walking on uneven terrain.

## 4. Discussion

In this paper, we proposed a systematic CPG-based control design scheme for bipedal walking robots based on the Japanese concept of Tegotae in Owaki et al. (2017) and Kano et al. (2019). To validate the proposed method, we designed hip, knee, and ankle joint controllers for a two-dimensional bipedal walking model. The results of dynamic simulations with the proposed bipedal walking model design have demonstrated that steady walking, stability, and spontaneous inter- and intra-limb coordination can be achieved. Furthermore, we found the model with three types of joint controllers to be highly adaptable to environmental changes during walking tasks. These findings imply that the systematic nature of the proposed control scheme can improve the motor function, i.e., adaptability, of bipedal walking robots.

We have previously shown the potential of the *Tegotae* approach in reproducing animals' locomotion and understanding the underlying mechanism based on the synthetic approaches. The *Tegotae* approach was first used by Owaki et al. (2017) to develop a minimal model for interlimb coordination on hexapod robot locomotion with CPG-based control, where all controllers were uniform for all elements. Kano et al. (2018) demonstrated gait transition between the concertina and scaffold-based locomotion on snake model simulation with reflex-like control, where all controllers were uniform, but generated non-rhythmic control signals. Kano et al. (2019) proposed detailed design of the *Tegotae* function, especially for motor command, using genetic algorithm (GA) to simulate a simple 1-D earthworm model with CPG-based control (uniform controllers for all elements). Compared to these approaches, here, we showed adaptive walking control on biped model with CPG and reflex-based control (non-uniform controllers, i.e., hip has CPG-based control, but the knee, and ankle have reflex-like controllers with no rhythmic signals). The novel attempts of this study can be summarised as follows: (i) First application of the *Tegotae* approach for the non-homogeneous system of animals' body, i.e., bipedal model with the hip, knee, and ankle joint, which need individual controllers for the generation of walking motion; (ii) combination between CPG-based rhythmic control for the hip joint and reflex-like non-rhythmic control for knee and ankle joints; finally, (iii) verification of adaptability against unknown environmental changes during bipedal walking.

The detail design guidelines of the *Tegotae* function for a local joint controller are as follows. The hips have periodic motions in which the swing leg descends forward and the stance leg kicks the ground alternately. For generating this motion, a phase oscillator is used as a controller for the hip joint (Equation 8). We used a heel load sensor, which reflects ground contact and load information during walking, and metatarsal and toe load sensors, for obtaining load information just before pushing-off the ground, as sensory information for the hip controllers. The feature of the *Tegotae* function of the hip joint is that we used the sensory information of not only the corresponding leg but also that of the other leg in designing the function. A mechanism called “Crossed Inhibitory Response,” which contributes to inter-limb coordination in bipedal walking, was reported in Stubbs and Mrachacz-Kersting (2009) and Gervasio et al. (2017). We also designed a *Tegotae* function using the load information of neighbouring legs in our hexapod model (Owaki et al., 2017). Based on the above considerations, we designed the *Tegotae* function of hips in Equation (9); when an action, e.g., −sinϕ_*i*_ > 0 for stance phase, and a reaction, e.g., $N_{h,i}^{V}>0$ for heel feels load, are highly consistent, the *Tegotae* function of the hip shows high value. See more details for the other three cases in Table 1.

**Table 1:** Design for Tegotae function.

**Joint**	**State**	***Action f*(*x*)**	***Reaction g*(*S*)**	***T*(*x, S*)**
Hip	Stance phase	−sinϕ_*i*_ > 0	$N_{h,i}^{V}>0$	$\left(-\text{sin}\phi_{i}\right)N_{h,i}^{V}$
		−sinϕ_*i*_ > 0	$N_{m,j}^{V}+N_{t,j}^{V}>0$	$\left(-\text{sin}\phi_{i}\right)\left(N_{m,j}^{V}+N_{t,j}^{V}\right)$
	Swing phase	sinϕ_*i*_ > 0	$N_{m,i}^{V}+N_{t,i}^{V}>0$	$\text{sin}\phi_{i}\left(N_{m,i}^{V}+N_{t,i}^{V}\right)$
		sinϕ_*i*_ > 0	$N_{h,j}^{V}>0$	$\text{sin}\phi_{i}N_{h,j}^{V}$
Knee	Stiff	χ_*i*_ > 0	$N_{i}^{V}>0$	$\chi_{i}N_{i}^{V}$
	Soft	−χ_*i*_ > 0	$N_{j}^{V}>0$	$\left(-\chi_{i}\right)N_{j}^{V}$
Ankle	Plantarflexion	ψ_*i*_ > 0	$N_{i}^{H}>0$	$\psi_{i}N_{i}^{H}$
	Dorsiflexion	−ψ_*i*_ > 0	$N_{j}^{V}>0$	$\left(-\psi_{i}\right)N_{j}^{V}$

The role of the knee joint during walking is important for stabilising the gait. During the stance phase, the knee joint stiffness is increased to support the body. In the swing phase, the knee joint stiffness must be dramatically reduced to realise efficient swinging of the swing leg. Therefore, the knee joint stiffness was adopted as a control variable. The sum of the heel, metatarsal, and toe loads at planter sensation was used as sensory information. The knee stiffness is switched ON and OFF to switch between the stance and swing phases. The conversion from the control variable to the joint stiffness was set up using the tanh and max function (Equation 11). As the dynamics of the control variable, a reflexive stiffness change based on non-periodic dynamics was modelled by the first order equation (Equation 12). The *Tegotae* function was designed as the product of the knee stiffness control variable and load on foot (heel, metatarsal, and toe). When the consistency between the action, e.g., χ_*i*_ > 0 for stiff knee, and reaction, e.g., $N_{i}^{V}>0$ for foot heels load, is high, the *Tegotae* function of the knee joint also takes a high value. The details for the other case are shown in Table 1.

The important functions of the ankle joint for gait stabilisation are to “push-off” (Lipfert et al., 2014; Zelik and Adamczyk, 2016) in the late stance phase and to suppress stumbling of the toe during the swing phase. Therefore, the control variables were set to indicate the non-periodic degree of plantar flexion and dorsiflexion of the ankle joint using the first order equation of Equation (17). The target angle of the ankle joint was set using the tanh function for the control variables (Equation 16). The horizontal GRFs of the foot $N_{i}^{H}$ were used as sensory information to generate propulsive force as the *Tegotae* function to express the push-off function in the first right-hand term of Equation (18). The *Tegotae* function for the dorsiflexion motion of the ankle joint during the swing phase was designed to adjust the degree of dorsiflexion according to the load of the foot of the other leg $N_{j}^{V}$ (see the details in Table 1).

In this research, various *Tegotae* functions have been selected and verified by trial and error in the design process. The *Tegotae* function used in this study is one of the examples that realised stable and adaptive walking. We can easily imagine that an inappropriate *Tegotae* function clearly does not lead to gait stabilisation. For example, consider a *Tegotae* function at the hip joint, where *f*(*x*) = sinϕ_*i*_ > 0, meaning swing phase, and $g\left(S\right)=N_{i}^{V}>0$ (foot feels load). This *Tegotae* function does not lead to a stable walking because of the inconsistency between action *f*(*x*) and reaction *g*(*S*). Thus, the point of designing the *Tegotae* function is to consider the physical consistency of the action and reaction for the desired motion, and to design the *Tegotae* function so that its value becomes large in such cases. Once such a *Tegotae* function is designed, it is possible to modify the control variables in a situation-dependent manner by modifying the control variables by increasing the *Tegotae* function as a feedback term ∂*T*(*x, S*)/∂*x*. Thus, the *Tegotae* approach enables the design of an autonomous decentralised controller in a systematic manner, by designing *Tegotae* function in line with the desired motions.

For the results of environmental adaptability on uneven terrain, Model 3, which has hip, knee, and ankle control, showed higher adaptability in a wide range of omega from low speed to high speed. However, in high speed walking, such as ω = 4.5 rad/s, Model 2 and Model 3 showed almost the same adaptability on the uneven terrain. The difference between Model 2 and Model 3 is the presence or absence of the hip controller based on Tegotae. In other words, Model 2 is a non-periodic reflex-based walking model without feedback for hip CPG (only feedforward CPG), whereas Model 3 implements Tegotae-based feedback for periodic CPG. Thus, when ω is small, i.e., slow speed walking, Tegotae feedback on CPG contributes to the adaptability of walking, whereas when ω is large, i.e., fast speed walking, the presence or absence of feedback to CPG does not affect the adaptability of walking. Manoonpong et al. (2007) showed that a walking controller based on a reflex model could achieve stable and fast walking, suggesting that the role of reflex-based control becomes salient in high-speed walking motions because the response time of feedback to CPG is not fast enough for the modification of the rhythmic control signals. In our model, Tegotae-based feedback to CPG at the hip joint and Tegotae-based feedback to reflex-based control at the knee and ankle joints were implemented. Therefore, the role of the feedback in the periodic and non-periodic controllers may have resulted in a high degree of adaptability to a wide range of ω. As shown in Figure 9B, Model 3 with PC shows high environmental adaptability to a wide range of ω.

In this study, we used plantar sensation (i.e., GRFs) as sensory information for feedback to CPG-based controllers. Past studies with humans and animals have shown that cutaneous receptors in the foot play an essential role in the control of gait (Dietz and Duysens, 2000; Duysens et al., 2000) and posture (Magnusson et al., 1990; Kavounoudias et al., 1998). For example, the reported effects of reducing plantar sensation by implementing an ice immersion technique (Nurse and Nigg, 2001; Eils et al., 2002; Elis et al., 2004) suggest that plantar sensation plays a critical role in gait modification. Similarly, various researchers have reported on the effects of impaired plantar sensation on gait plasticity due to ageing (Sorock and Labiner, 1992), diseases, such as diabetes mellitus (Cavanagh et al., 1993), or congenital insensitivity to pain with anhidrosis (Zhang et al., 2013; Yozu et al., 2016). Decreased tactile sensation, with ageing-related impaired sensory function in limbs, has been reported to lead to elderly falling accidents (Sorock and Labiner, 1992). Additionally, patients with diabetic neuropathy, which is commonly associated with damage to nerves in the feet, have been reported to have significantly impaired control of gait and posture (Cavanagh et al., 1993). Thus, our results, which demonstrate that implementing a plantar sensory feedback mechanism in a systematic control scheme improved adaptability and walking stability, are consistent with the findings of previous human and animal studies on the influence of plantar sensation on gait and postural control.

The realisation of adaptive bipedal walking is known to be dependent on the generation of a limit cycle in the state space, which comprises a brain-nervous system (i.e., the control system), musculoskeletal system (i.e., the mechanical system), and environment (Taga et al., 1991; Taga, 1994, 1995). For robots, the structural stability provided by a limit cycle affords robustness against environmental perturbations. However, design principles that can be applied to concretely establish a limit cycle with a large basin of attraction have yet to be conceptualised. One significant reason for this is that not enough sensory information is fed back to the control system in a limit cycle. Thus, sensory-motor coordination, which refers to the condition that movement induces sensory stimulation, which in turn influences the movement, must be considered to generate a more stable limit cycle (Pfeifer and Bongard, 2006; Pfeifer et al., 2007). Considering this, we must focus on the deformability of the underlying soft body of a robot. A soft body allows a robot to stabilise its motion as it extracts various types of sensory information; this is possible because it is flexible enough to deform to maintain stability during movement, resulting in a close relationship between motion and perception. Human soles are considered to be relatively soft. As a human walks, their relatively soft feet come into direct contact with the environment; the deformability of the feet, i.e., the softness of the sole and mobility of the joints of the feet, allows them to conform to the ground surface, enabling the extraction of diverse sensory information. Thus, the soft deformability of the foot of our bipedal walking model is also believed to have contributed to the high adaptability and stability observed in our results.

It should be noted that the phase-modulation mechanism underlying the proposed model is significantly different from that of any previous model, e.g., the previously reported phase-reset scheme (Tsujita et al., 2003; Aoi and Tsuchiya, 2005, 2006; Aoi et al., 2010). The phase-reset scheme, which entails resetting the phase of the oscillator to zero once the foot makes contact with the ground, only utilises qualitative information about the status of contact between the foot and ground (i.e., on or off). In contrast, our design methodology uses quantitative information that describes the extent to which each foot “feels” the GRF, representing the sensory information resulting from the deformation of soft feet. This was possible because highly adaptive behaviours emerge in response to environmental changes, and we were able to exploit these behaviours in our proposed design. Nevertheless, this study has several limitations. First, we only modelled a two-dimensional walking robot in the sagittal plane. This is because we wanted to focus on evaluating and validating our control scheme. Secondly, we utilised actuators, i.e., PD-based servo motors, as the model for each joint. Humans have antagonistic muscles that generate joint torques that allow us to exploit mono-articular and biarticular muscles for motion generation. This difference will be the focus of future work as we plan to design a more complex model. Lastly, the proposed model needs to be validated by performing real-world experiments; this is also a focus of future studies.

## Data Availability Statement

The original contributions presented in the study are included in the article/Supplementary Material, further inquiries can be directed to the corresponding author/s.

## Author Contributions

DO, JN, and AI conceived the research idea and performed data collection. SH designed the model and controllers and conducted the simulations. SH and DO conducted the analysis. All authors participated equally in the preparation of this manuscript.

## Conflict of Interest

The authors declare that the research was conducted in the absence of any commercial or financial relationships that could be construed as a potential conflict of interest.
