# Supplementary material for: Tegotae-Based Control Produces Adaptive Inter- and Intra-limb Coordination in Bipedal Walking
Source: Front Neurorobot. 2021 May 12;15:629595. doi: 10.3389/fnbot.2021.629595 (PMC8149599; doi:10.3389/fnbot.2021.629595)
Supplement: Supplementary file 3 [file Data_Sheet_1.PDF]

Supplementary Material

1 EQUATION OF MOTION FOR 2 DIMENSIONAL BIPEDAL WALKING MODEL

The equation of motion for each mass that consists of the 2-D bipedal walking model is described as

$$
\begin{aligned}
m_{body}\,\ddot{\boldsymbol{r}}_{body} + \boldsymbol{g}(\boldsymbol{r}_{body}) &= \boldsymbol{f}_{link,body} + \boldsymbol{f}_{actuator,body}, && \text{(S1)}\\
m_{trunk}\,\ddot{\boldsymbol{r}}_{trunk} + \boldsymbol{g}(\boldsymbol{r}_{trunk}) &= \boldsymbol{f}_{link,trunk} + \boldsymbol{f}_{actuator,trunk}, && \text{(S2)}\\
m_{hip}\,\ddot{\boldsymbol{r}}_{hip} + \boldsymbol{g}(\boldsymbol{r}_{hip}) &= \boldsymbol{f}_{link,hip} + \boldsymbol{f}_{actuator,hip}, && \text{(S3)}\\
m_{knee}\,\ddot{\boldsymbol{r}}_{knee_L} + \boldsymbol{g}(\boldsymbol{r}_{knee_L}) &= \boldsymbol{f}_{link,knee_L} + \boldsymbol{f}_{actuator,knee_L}, && \text{(S4)}\\
m_{knee}\,\ddot{\boldsymbol{r}}_{knee_R} + \boldsymbol{g}(\boldsymbol{r}_{knee_R}) &= \boldsymbol{f}_{link,knee_R} + \boldsymbol{f}_{actuator,knee_R}, && \text{(S5)}\\
m_{ankle}\,\ddot{\boldsymbol{r}}_{ankle_L} + \boldsymbol{g}(\boldsymbol{r}_{ankle_L}) &= \boldsymbol{f}_{link,ankle_L} + \boldsymbol{f}_{actuator,ankle_L} + \boldsymbol{f}_{spring,ankle_L}, && \text{(S6)}\\
m_{ankle}\,\ddot{\boldsymbol{r}}_{ankle_R} + \boldsymbol{g}(\boldsymbol{r}_{ankle_R}) &= \boldsymbol{f}_{link,ankle_R} + \boldsymbol{f}_{actuator,ankle_R} + \boldsymbol{f}_{spring,ankle_R}, && \text{(S7)}\\
m_{heel}\,\ddot{\boldsymbol{r}}_{heel_L} + \boldsymbol{g}(\boldsymbol{r}_{heel_L}) &= \boldsymbol{f}_{link,heel_L} + \boldsymbol{f}_{actuator,heel_L} + \boldsymbol{f}_{spring,heel_L} + \boldsymbol{f}_{ground,heel_L}, && \text{(S8)}\\
m_{heel}\,\ddot{\boldsymbol{r}}_{heel_R} + \boldsymbol{g}(\boldsymbol{r}_{heel_R}) &= \boldsymbol{f}_{link,heel_R} + \boldsymbol{f}_{actuator,heel_R} + \boldsymbol{f}_{spring,heel_R} + \boldsymbol{f}_{ground,heel_R}, && \text{(S9)}\\
m_{mid}\,\ddot{\boldsymbol{r}}_{mid_L} + \boldsymbol{g}(\boldsymbol{r}_{mid_L}) &= \boldsymbol{f}_{link,mid_L} + \boldsymbol{f}_{actuator,mid_L} + \boldsymbol{f}_{spring,mid_L} + \boldsymbol{f}_{ground,mid_L}, && \text{(S10)}\\
m_{mid}\,\ddot{\boldsymbol{r}}_{mid_R} + \boldsymbol{g}(\boldsymbol{r}_{mid_R}) &= \boldsymbol{f}_{link,mid_R} + \boldsymbol{f}_{actuator,mid_R} + \boldsymbol{f}_{spring,mid_L} + \boldsymbol{f}_{ground,mid_R}, && \text{(S11)}\\
m_{toe}\,\ddot{\boldsymbol{r}}_{toe_L} + \boldsymbol{g}(\boldsymbol{r}_{toe_L}) &= \boldsymbol{f}_{link,toe_L} + \boldsymbol{f}_{actuator,toe_L} + \boldsymbol{f}_{spring,toe_L} + \boldsymbol{f}_{ground,toe_L}, && \text{(S12)}\\
m_{toe}\,\ddot{\boldsymbol{r}}_{toe_R} + \boldsymbol{g}(\boldsymbol{r}_{toe_R}) &= \boldsymbol{f}_{link,toe_R} + \boldsymbol{f}_{actuator,toe_R} + \boldsymbol{f}_{spring,toe_R} + \boldsymbol{f}_{ground,toe_R}, && \text{(S13)}\\
& && \text{(S14)}
\end{aligned}
$$

where $\boldsymbol{r}_i$ represents the position vector of each mass point on the 2-D saggitale plane. $\boldsymbol{g}(\boldsymbol{r}_i) = m_i g$ are the gravitational force for each mass. $\boldsymbol{f}_{link,i}$, $\boldsymbol{f}_{actuator,i}$, $\boldsymbol{f}_{spring,i}$, and $\boldsymbol{f}_{ground,i}$ represent the sum of forces from link, joint actuator, and passive spring at the toe joints, and force applied from ground, respectively. The suffix i denotes the name of the mass point.

1.1 Mass-link System

For the sake of understanding, we here consider a simple model of a mass-link system as shown Fig. S1, and describe the forces $\boldsymbol{f}_{link,i}$ generated by links modelled by "rigid" linear springs and dampers, and the forces $\boldsymbol{f}_{actuator,i}(\boldsymbol{f}_{spring,i})$ generated by torques from joint actuators (or rotational springs and dampers).

The force from spring and damper of the ith rigid link is described by the following equation:

$$f_{s,i} = K_i(l_i - \bar{l}_i) + C_i\dot{l}_i, \qquad \text{(S15)}$$

where K_i, C_i are the coefficients of the spring and damper at the i link. $l_i, \dot{l}_i$ are the length of the link and time derivative of the link length, respectively. The ith link connects the masses $\boldsymbol{r}_{i-1}$ and $\boldsymbol{r}_i$, then, the length is described as $l_i = |\boldsymbol{r}_{i-1} - \boldsymbol{r}_i|$. The unit vector $\boldsymbol{t}_i$ parallel to the ith link is described as $(\boldsymbol{r}_i - \boldsymbol{r}_{i+1})/l_i$. The sum of forces applied to the ith mass point from links' forces is described by using

the forces from $i-1$ and i links,

$$\begin{aligned} \boldsymbol{f}_{link,i} &= f_{s,i}\boldsymbol{t}_i - f_{s,i-1}\boldsymbol{t}_{i-1}, \quad \text{(S16)} \\ &= f_{s,i}\frac{\boldsymbol{r}_i - \boldsymbol{r}_{i+1}}{l_i} - f_{s,i-1}\frac{\boldsymbol{r}_{i-1} - \boldsymbol{r}_i}{l_{i-1}} \quad \text{(S17)} \end{aligned}$$

The unit vector $\boldsymbol{n}_i$ perpendicular to the ith link is described by using rotational matrix as follows:

$$\boldsymbol{n}_i = \begin{bmatrix} \cos(\pi/2) & -\sin(\pi/2) \\ \sin(\pi/2) & \cos(\pi/2) \end{bmatrix} \boldsymbol{t}_i = \begin{bmatrix} 0 & -1 \\ 1 & 0 \end{bmatrix} \boldsymbol{t}_i \quad \text{(S18)}$$

The sum of forces applied to the ith mass point from joint actuators (or passive springs) is described as follows:

$$\begin{aligned} \boldsymbol{f}_{actuator,i} &= \frac{\tau_{a,i-1} - \tau_{a,i}}{l_{i-1}}\boldsymbol{n}_{i-1} + \frac{\tau_{a,i+1} - \tau_{a,i}}{l_i}\boldsymbol{n}_i, \quad \text{(S19)} \\ \boldsymbol{f}_{spring,i} &= \frac{\tau_{s,i-1} - \tau_{s,i}}{l_{i-1}}\boldsymbol{n}_{i-1} + \frac{\tau_{s,i+1} - \tau_{s,i}}{l_i}\boldsymbol{n}_i, \quad \text{(S20)} \end{aligned}$$

where $\tau_{a,i}$ and $\tau_{s,i}$ are the torque by an actuator and passive spring at the ith joint. If there are no mass point or actuators/passive spring at the $(i-1)$th or $(i+1)$th neighbouring joint, the value is zero.

$\tau_{a,i}$ is described in the equations (1)–(4) in the main text. $\tau_{s,i}$ is modelled as rotational springs and dampers by the joint angle θ_i and joint angular velocity $\dot{\theta}_i$ as follows:

$$\tau_{s,i} = k_i\theta_i + c_i\dot{\theta}_i, \quad \text{(S21)}$$

where k_i, c_i are the coefficients of the passive spring and damper.

1.2 Ground model

The contact with the ground was also modelled as a simple spring-damper system. Here, for simplicity, we describe the model in the case of a flat ground. When the vertical position y_i of the heels, mids, and toes become less than 0, $y_i < 0$, vertical force from the ground were applied to the points as described by the following equations:

$$N_i^v = \begin{cases} -k_{ground}\, y_i - c_{ground}\, \dot{y}_i & (y_i < 0) \\ 0 & (y_i >= 0) \end{cases} \quad \text{(S22)}$$

where k_{ground}, c_{ground} are the coefficients of the passive spring and damper, respectively. The horizontal force from the ground was modelled by Coulomb and viscous friction for simplicity:

$$N_i^h = -\mu_c \mathrm{sign}(\dot{x}_i) N_i^v - \mu_v \dot{x}_i, \quad \text{(S23)}$$

where μ_c and μ_v denote the coefficient of Coulomb and viscous friction, respectively. Here, we omit the static friction.

In sum, the force from the flat ground was described by

$$\boldsymbol{f}_{ground,i} = \begin{bmatrix} N_i^h \\ N_i^v \end{bmatrix} \tag{S24}$$

In the case of uneven terrain, the ground reaction forces are modelled as acting vertically and horizontally relative to the circle ground.

1.3 Simulation Environment

The simulation program was written in C++ language. The differential equations were integrated using the fourth-order Runge–Kutta method with a time step of 1.00×10^{-4} s.

2 MEASUREMENT OF HUMAN WALKING

We enrolled one healthy subject for measuring normal human walking. The participant provided written informed consent before the data collection, and the study was approved by the institutional review board.

The subject was asked to walk 7 m over two to ten trials. The subject was instructed to walk at a self-selected comfortable pace. The results consist of an average of more than five strides during the successful trials. In addition, using adhesive tape, 33 reflective markers were attached to 12 segments. For all the measurements, the MAC 3D System (120 Hz; Motion Analysis Corporation, Santa Rosa, CA, USA) was used to measure the coordinates of each reflective marker. The ground reaction force data were obtained at a 1200-Hz sampling rate using four 90 cm$\times$60 cm force plates (Anima Corporation, Chofu, Tokyo, Japan). The three-dimensional coordinates and ground reaction force data were smoothed using a bidirectional fourth-order Butterworth low-pass filter with cut-off frequencies of 20 Hz and 200 Hz, respectively. This study used a 12-segment model based on anthropometric data, in accordance with the work of Dumas S(1), which consisted of the feet, shanks, thighs, pelvis, thorax, upper arms, and forearms. For each joint in the lower extremities, the kinematic data were calculated using a joint coordinate system S(2). In addition, the kinetics of the lower extremity joints were estimated using inverse dynamics S(3). All kinematic and kinetic data were time-normalized to 100$\%$ of the one gait cycle. The kinetic data were normalized to the patient's body weight. The parameters were calculated using a customized software program created with MATLAB (Mathworks Inc., Natick, MA, USA).

3 SUPPLEMENTARY TABLES AND FIGURE

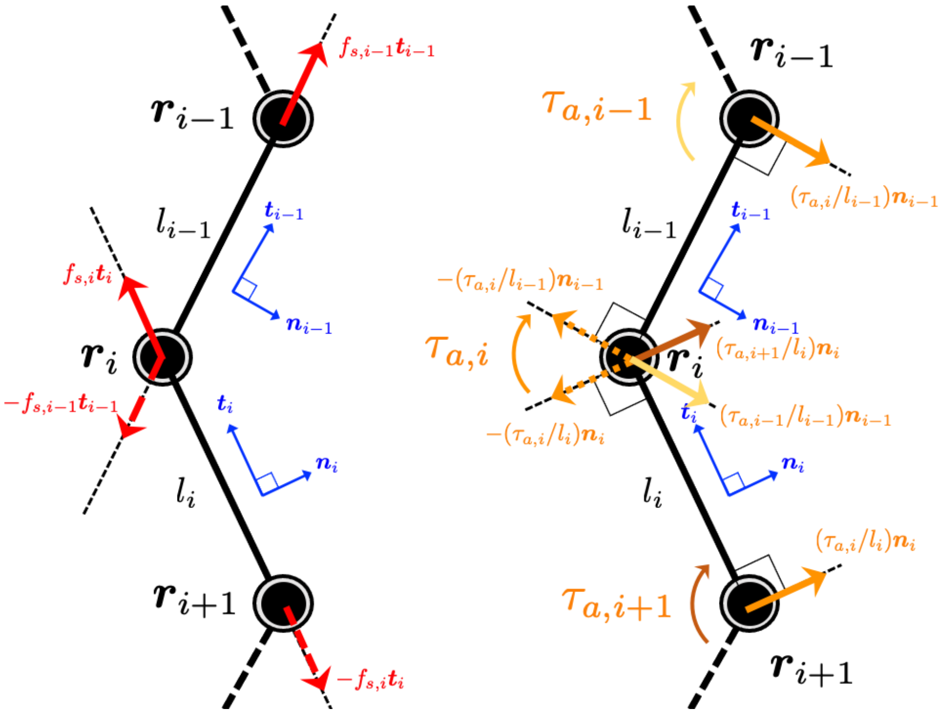

Figure S1. Schematic of mass-link model.

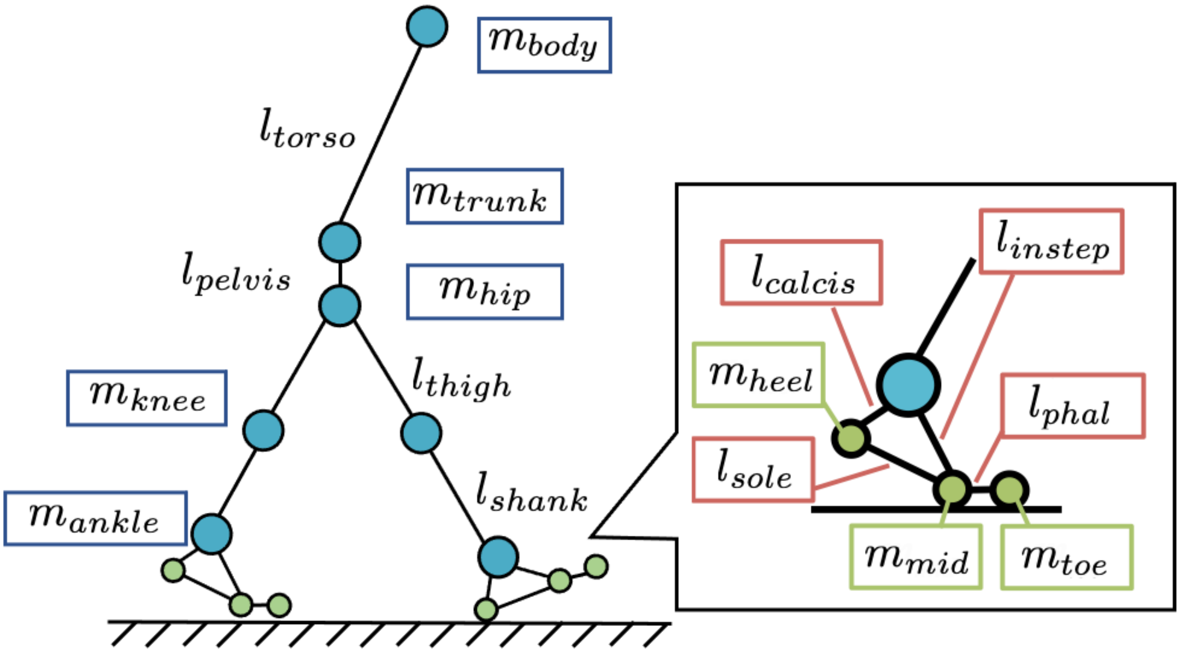

Figure S2. Defition of body parameters.

REFERENCES

[1]R. Dumas, L. Cheze, J.P. Verriest, “Adjustments to McConville et al. and Young et al. body segment inertial parameters,” *J Biomech.*, vol. 40, no. 3, pp. 543-53, 2007.

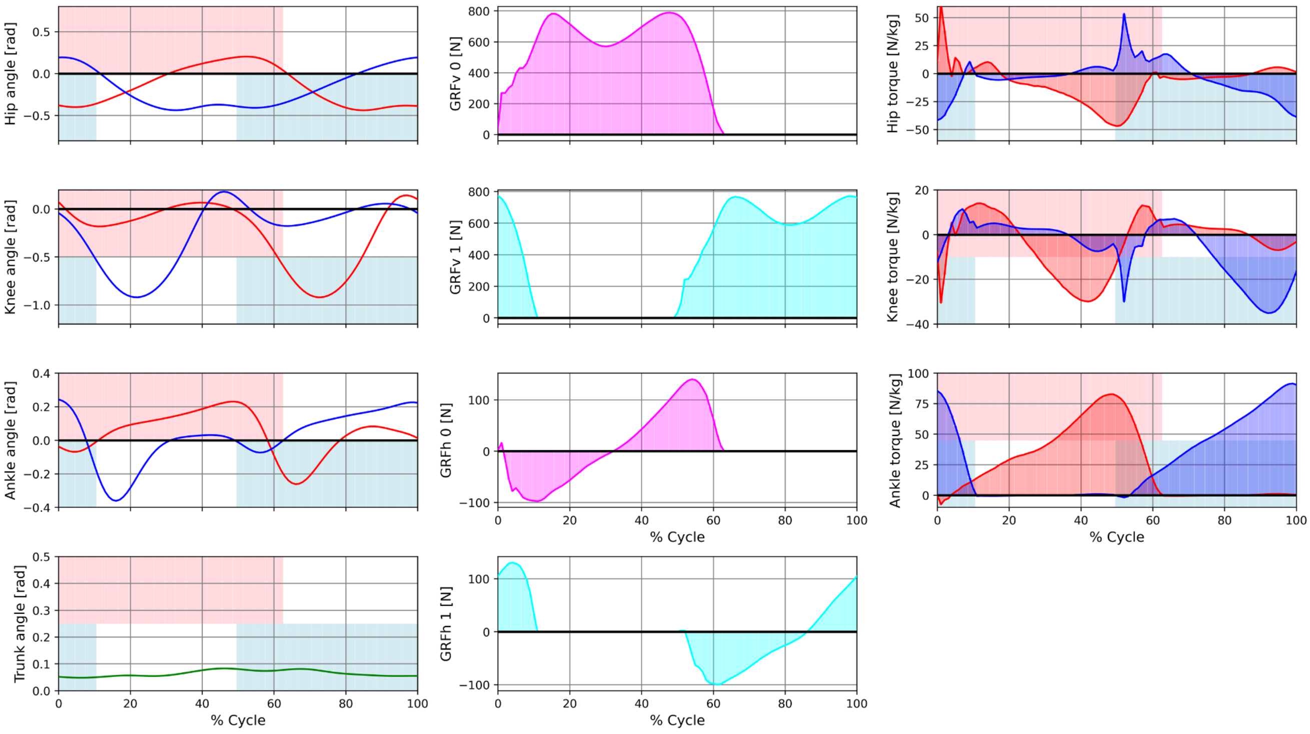

Figure S3. Time series data of the steady walking in human (a health subject). Left panels show the hip, knee, ankle, and trunk angle from top to bottom. The red and blue lines show the left and right legs, respectively. The centre panels show vertical (top, second) and horizontal (third, bottom) ground reaction forces (GRFs). The magenta and cyan coloured regions represent the forces on the left and right legs, respectively. The right panels represent the torque applied to the hip, knee, ankle joints. The red, blue, and green colour regions represent the left, right legs, and trunk respectively. For the left and right panels, the pink and sky blue coloured regions represent the stance phase of left and right legs, which are determined by the vertical GRFs. The data was measured with the help of Dr. Yusuke Sekiguchi (Physical Therapist: PT) and Dr. Keita Honda (PT).

[2]D.A. Winter, editor., “Biomechanics and motor control of human movement. fourth ed. Hoboken, New Jersey: John Wiley & Sons, Inc., 2009.

[3]G.E. Robertson, G.E. Caldwell, J. Hamill, G. Kamen, S. Whittlesey, “Research methods in biomechanics: *Human kinetics*; 2013.

[4]M. Wisse, J. van Frankenhuyzen “Design and construction of mike: A 2D autonomous biped based on passive dynamic walking” In Proc. of the Second International Symposium on Adaptive Motion of Animals and Machines (AMAM2003), 2003.

[5]https://www.agilityrobotics.com

Table S1. Body and enviromental parameters in simulations

Environmental Parameters		Values
dt	time step	0.0001[s]
g	gravitational acceleration	$9.81[\mathrm{m/s^2}]$
k_{ground}	elasticity of the ground	1000000[N/m]
c_{ground}	viscosity of the ground	500[Ns/m]
μ_c	coefficient of Coulomb friction	1.0
μ_v	coefficient of viscous friction	2000[Ns/m]
Body Parameters		Values
m_{body}	weight of body	37.184[kg]
m_{trunk}	weight of trunk	6.6374[kg]
m_{hip}	weight of hip	6.6374[kg]
m_{knee}	weight of knee	4.5096[kg]
m_{ankle}	weight of ankle	1.136[kg]
m_{heel}	weight of heel	0.534[kg]
m_{mid}	weight of mid	0.2[kg]
m_{toe}	weight of toe	0.156[kg]
k	elasticity of links	1000000[N/m]
k_{pelvis}	elasticity of pelvis	500000[N/m]
k_{calcis}	elasticity of calcis	500000[N/m]
k_{instep}	elasticity of instep	500000[N/m]
c	viscosity of links	500[Ns/m]
c_{pelvis}	viscosity of pelvis	2000[Ns/m]
c_{calcis}	viscosity of calcis	2000[Ns/m]
c_{insetp}	viscosity of instep	2000[Ns/m]
l_{torso}	length of torso	0.492[m]
l_{pelvis}	length of pelvis	0.05[m]
l_{thigh}	length of thigh	0.383[m]
l_{shank}	length of shank	0.407[m]
l_{calcis}	length of calcis	0.0849[m]
l_{instep}	length of instep	0.1145[m]
l_{sole}	length of sole	0.1575[m]
l_{phal}	length of phal	0.0525[m]

Table S2. Control parameters in simulations

Control Parameters		Values
K_{hip}	proportional gain of hip actuator	500[Nm/rad]
D_{hip}	differential gain of hip actuator	10[Nm/rad s]
$C_{1,hip}$	amplitude of leg swing	0.42 [rad]
$C_{2,hip}$	offset angle of leg swing	0[rad]
ω	intrinsic anglar velocity	4[rad/s]
$\sigma_{hip,1}$	feedback gain for hip actuator	0.0004
$\sigma_{hip,2}$	feedback gain for hip actuator	0.0005
D_{knee}	differential gain of knee actuator	1[Nm/rad s]
$C_{1,knee}$	range of proportional gain of knee actuator	1000[Nm/rad]
$C_{2,knee}$	offset of proportional gain of knee actuator	15[Nm/rad]
c_{knee}	response time for the first-order equation	20
$\sigma_{knee,1}$	feedback gain for knee actuator	0.05
$\sigma_{knee,2}$	feedback gain for knee actuator	0.05
K_{ankle}	proportional gain of ankle actuator	80[Nm/rad s]
D_{ankle}	differential gain of ankle actuator	10[Nm/rad s]
$C_{1,ankle}$	amplitude of foot flexion	1.0 [rad]
$C_{2,ankle}$	offset angle of foot flexion	0.0[rad]
c_{ankle}	response time for the first-order equation	5
$\sigma_{ankle,1}$	feedback gain for ankle actuator	0.001
$\sigma_{ankle,2}$	feedback gain for ankle actuator	0.001

Table S3. Comparison of speed and Froude number with walking robots and human

	Leg length l [m]	speed v [m/s]	Relative speed v/l [/s]	Frounde number v^2/gl
Passive walker S(4)	0.8	0.6	0.7	0.05
Mike S(4)	0.75	0.4	0.5	0.02
RunBot (Manoonpong et al. (2007))	0.23	0.8	3.48	0.28
Cassie S(5)	1.15	1.38 (5km/h)	1.207	0.1709
Model 3 with PC	0.79	0.8177 – 0.9735	1.035 – 1.232	0.08629 – 0.1223
human (Fig.S3)	0.8051	1.0293	1.2784	0.1414
